# Monte Carlo Planning Method Estimates Planning Horizons during Interactive Social Exchange

**DOI:** 10.1371/journal.pcbi.1004254

**Published:** 2015-06-08

**Authors:** Andreas Hula, P. Read Montague, Peter Dayan

**Affiliations:** 1 Wellcome Trust Centre for Neuroimaging, University College London, London, United Kingdom; 2 Wellcome Trust Centre for Neuroimaging, University College London, London, United Kingdom; 3 Human Neuroimaging Laboratory, Virginia Tech Carilion Research Institute, Roanoke, Virginia, United States of America; 4 Department of Physics, Virginia Polytechnic Institute and State University, Blacksburg, Virginia, United States of America; 5 Gatsby Computational Neuroscience Unit, University College London, London, United Kingdom; Massachusetts Institute of Technology, UNITED STATES

## Abstract

Reciprocating interactions represent a central feature of all human exchanges. They have been the target of various recent experiments, with healthy participants and psychiatric populations engaging as dyads in multi-round exchanges such as a repeated trust task. Behaviour in such exchanges involves complexities related to each agent’s preference for equity with their partner, beliefs about the partner’s appetite for equity, beliefs about the partner’s model of their partner, and so on. Agents may also plan different numbers of steps into the future. Providing a computationally precise account of the behaviour is an essential step towards understanding what underlies choices. A natural framework for this is that of an interactive partially observable Markov decision process (IPOMDP). However, the various complexities make IPOMDPs inordinately computationally challenging. Here, we show how to approximate the solution for the multi-round trust task using a variant of the Monte-Carlo tree search algorithm. We demonstrate that the algorithm is efficient and effective, and therefore can be used to invert observations of behavioural choices. We use generated behaviour to elucidate the richness and sophistication of interactive inference.

## Author Summary

Agents interacting in games with multiple rounds must model their partner’s thought processes over extended time horizons. This poses a substantial computational challenge that has restricted previous behavioural analyses. By taking advantage of recent advances in algorithms for planning in the face of uncertainty, we demonstrate how these formal methods can be extended. We use a well studied social exchange game called the trust task to illustrate the power of our method, showing how agents with particular cognitive and social characteristics can be expected to interact, and how to infer the properties of individuals from observing their behaviour.

This is a *PLOS Computational Biology* Methods paper.

## Introduction

Successful social interactions require individuals to understand the consequences of their actions on the future actions and beliefs of those around them. To map these processes is a complex challenge in at least three different ways. The first is that other peoples’ preferences or utilities are not known exactly. Even if the various components of the utility functions are held in common, the actual values of the parameters of partners, e.g., their degrees of envy or guilt [1–6], could well differ. This ignorance decreases through experience, and can be modeled using the framework of a partially observable Markov decision process (POMDP). However, normal mechanisms for learning in POMDPs involve probing or running experiments, which has the potential cost of partners fooling each other. The second complexity is represented by characterizing the form of the model agents have of others. In principle, agent A’s model of agent B should include agent B’s model of agent A; and in turn, agent B’s model of agent A’s model of agent B, and so forth. The beautiful theory of Nash equilibria [7], extended to the case of incomplete information via so-called Bayes-Nash equilibria [8] dispenses with this so-called cognitive hierarchy [9–12], looking instead for an equilibrium solution. However, a wealth of work (see for instance [13]) has shown that people deviate from Nash behaviour. It has instead been proposed that people model others to a strictly limited, yet non-negligible, degree [9, 12].

The final complexity arises when we consider that although it is common in experimental economics to create one-shot interactions, many of the most interesting and richest aspects of behaviour arise with multiple rounds of interactions. Here, for concreteness, we consider the multi round trust task, which is a social exchange game that has been used with hundreds of pairs (dyads) of subjects, including both normal and clinical populations [14–18]. This game has been used to show that characteristics that only arise in multi-round interactions such as defection (agent A increases their cooperation between two rounds; agent B responds by decreasing theirs) have observable neural consequences that can be measured using functional magnetic resonance imaging (fMRI) [16, 19–22].

The interactive POMDP (IPOMDP) [23] is a theoretical framework that formalizes many of these complexities. It characterizes the uncertainties about the utility functions and planning over multiple rounds in terms of a POMDP, and constructs an explicit cognitive hierarchy of models about the other (hence the moniker ‘interactive’). This framework has previously been used with data from the multi-round trust task [22, 24]. However, solving IPOMDPs is computationally extremely challenging, restricting those previous investigations to a rather minuscule degree of forward planning (just two- out of what is actually a ten-round interaction). Our main contribution is the adaptation of an efficient Monte Carlo tree search method, called partially observable Monte Carlo planning (POMCP) to IPOMDP problems. Our second contribution is to illustrate this algorithm through examination of the multiround trust task. We show characteristic patterns of behaviour to be expected for subjects with particular degrees of inequality aversion, other-modeling and planning capacities, and consider how to invert observed behaviour to make inferences about the nature of subjects’ reasoning capacities.

## Results

We first briefly review Markov decision processes (MDPs), their partially observable extensions (POMDPs), and the POMCP algorithm invented to solve them approximately, but efficiently. These concern single agents. We then discuss IPOMDPs and the application of POMCP to solving them when there are multiple agents. Finally, we describe the multi-round trust task.

### Partially Observable Markov Decision Processes

**Fig 1:**
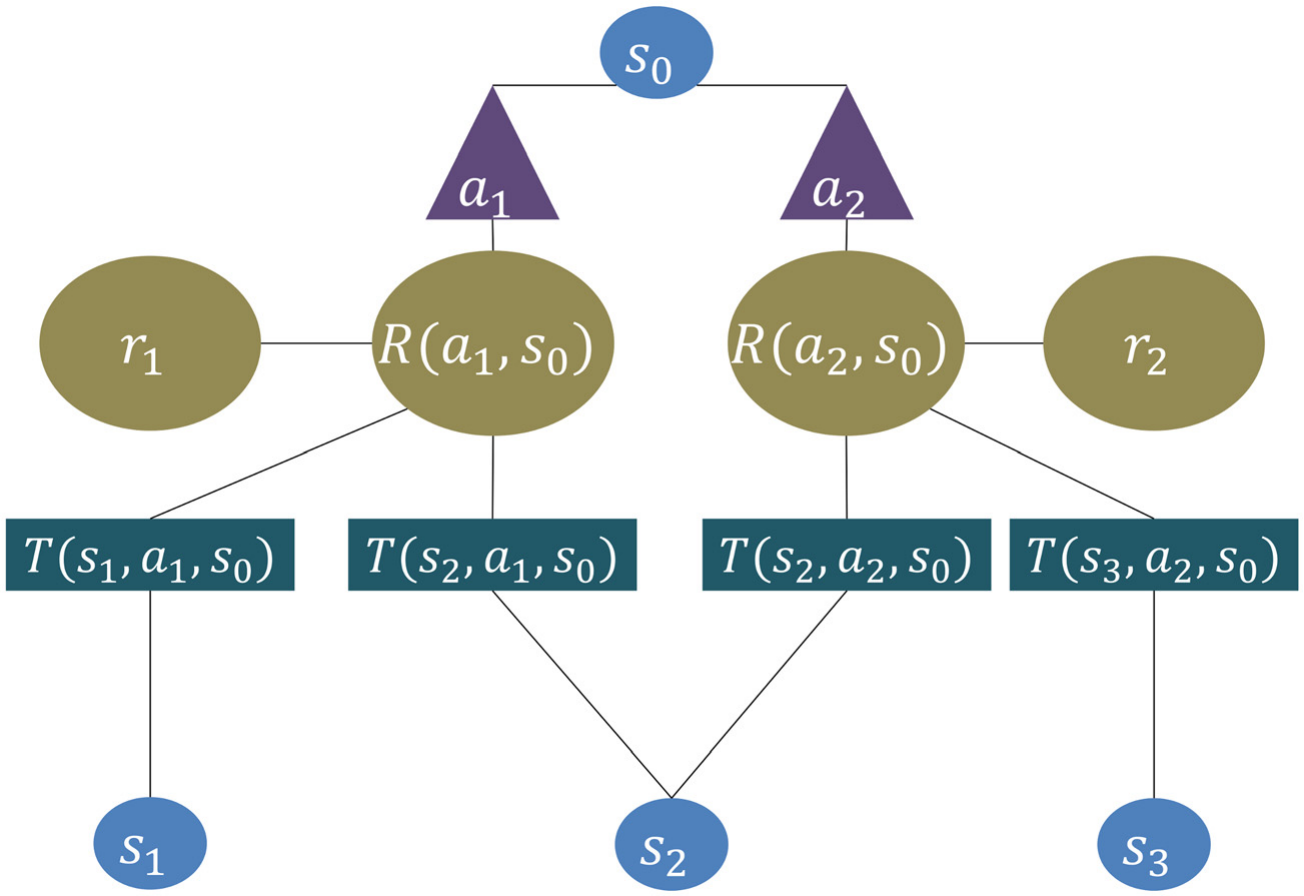
A Markov decision process. The agent starts at state *s*_0_ and has two possible actions *a*_1_ and *a*_2_. Exercising either, it can transition into three possible states, one of which (*s*_2_) can be reached through either action. Each state and action combination is associated with a particular reward expectation *R*(*a*, *s*). Based on this information, the agent can choose an action and transitions with probability 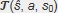 to a new state *ŝ*, obtaining an actual reward *r* in the process. The procedure is then repeated from the new state, with its’ given action possibilities or else the decision process might end, depending on the given process.

A Markov decision process (MDP) [25] is defined by sets 𝓢 of “states” and 𝓐 of “actions”, and several components that evaluate and link the two, including transition probabilities 𝓣, and information ℛ about possible rewards. States describe the position of the agent in the environment, and determine which actions can be taken, accounting for, at least probabilistically, the consequences for rewards and future states. Transitions between states are described by means of a collection of transition probabilities 𝓣, assigning to each possible state *s* ∈ 𝓢 and each possible action *a* ∈ 𝓐 from that state, a transition probability distribution or measure ${𝓣}_{s\hat{s}}^{a}=𝓣(\hat{s},a,s):=\mathbb{P}[\hat{s}|s,a]$ which encodes the likelihood of ending in state $\hat{s}$ after taking action *a* from state *s*. The Markov property requires that the transition (and reward probabilities) only depend on the current state (and action), and are independent from the past events. An illustration of these concepts can be found in Fig 1.

**Fig 2:**
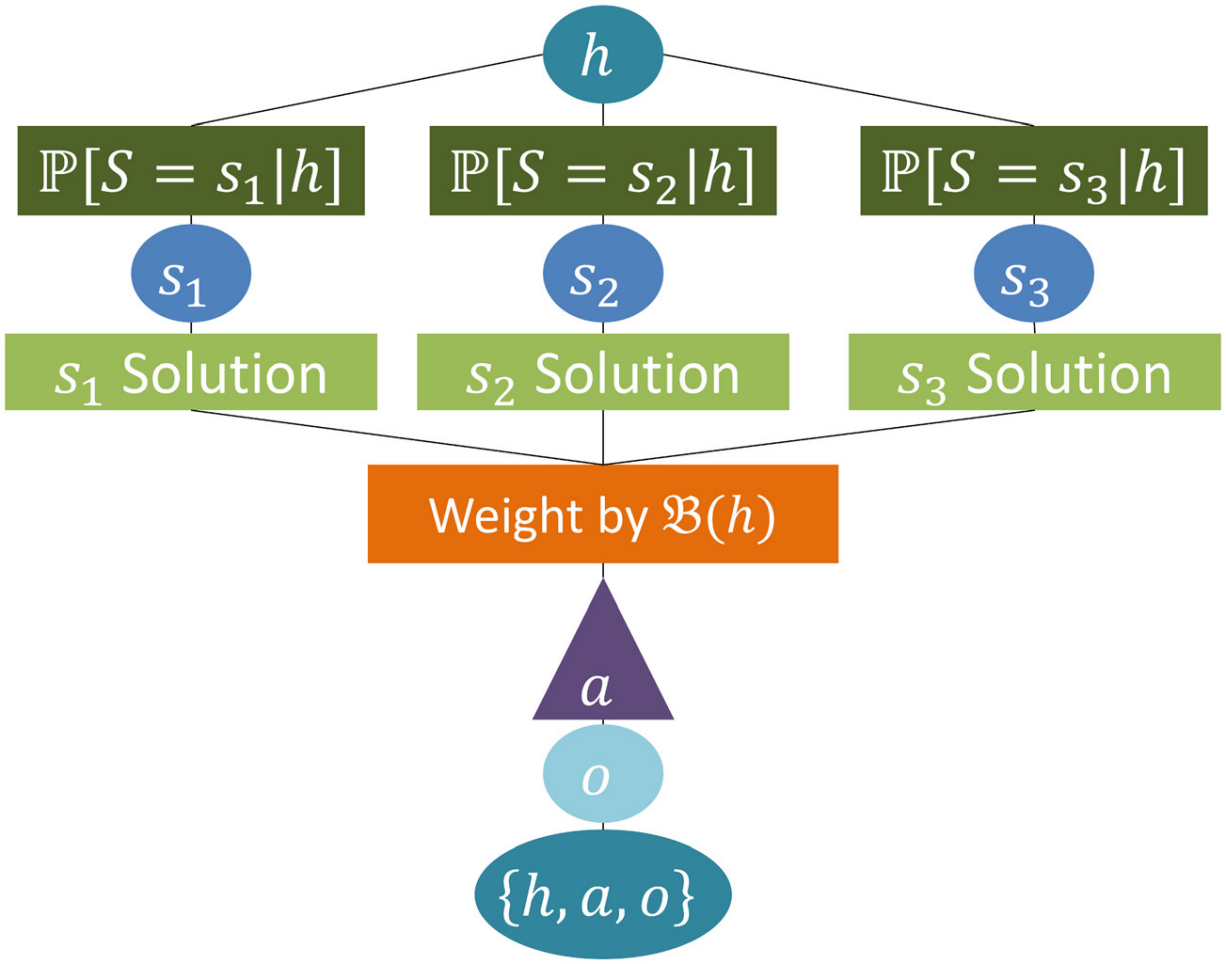
A partially observable Markov decision process. Starting from a observed interaction history *h*, the agents use their belief state 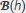, to determine how likely they are to find themselves in one of three possible actual states *s*_1_, *s*_2_, *s*_3_. The POMDP solution requires to integrate over all possible states according to the belief state at every possible following history. The solution allows to choose the next action a. Following this, an observation *o* is obtained by the agent and the new history {*h*, *a*, *o*} becomes the starting point for the next decision.

By contrast, in a partially observable MDP (i.e., a POMDP [26]), the agent can also be uncertain about its state *s*. Instead, there is a set of observations *o* ∈ 𝓞 that incompletely pin down states, depending on the observation probabilities ${𝓦}_{\hat{s}o}^{a}=𝓦(o,a,\hat{s}):=\mathbb{P}[o|\hat{s},a].$ These report the probability of observing *o* when action *a* has occasioned a transition to state $\hat{s}$. See Fig 2 for an illustration of the concept.

We use the notation *s*_*t*_ = *s*, *a*_*t*_ = *a* or *o*_*t*_ = *o* to refer explicitly to the outcome state, action or observation at a given time. The *history* *h* ∈ ℋ is the sequence of actions and observations, wherein each action from the point of view of the agent moves the time index ahead by 1, *h*_*t*_: = {*o*_0_, *a*_0_, *o*_1_, *a*_1_, …, *a*_*t*−1_, *o*_*t*_}. Here *o*_0_ may be trivial (deterministic or empty). The agent can perform Bayesian inference to turn its history at time *t* into a distribution ℙ[*S*_*t*_ = *s*_*t*_∣*h*_*t*_] over its state at time *t*, where *S*_*t*_ denotes the random variable encoding the uncertainty about the current state at time *t*. This distribution is called its belief state 𝓑(*h*_*t*_), with ℙ_𝓑(*h*_*t*_)_[*S*_*t*_ = *s*_*t*_]: = ℙ[*S*_*t*_ = *s*_*t*_∣*h*_*t*_]. Inference depends on knowing 𝓣, 𝓦 and the distribution over the initial state *S*_0_, which we write as 𝓑(*h*_0_). Information about rewards ℛ comprises a collection of utility functions *r* ∈ ℛ, *r*:𝓐 × 𝓢 × 𝓞 → ℝ, a discount function Γ ∈ ℛ,Γ:ℕ → [0, 1] and a survival function *H* ∈ ℛ, *H*:ℕ × ℕ → [0, 1]. The utility functions determine the immediate gain associated with executing action *a* at state *s* and observing *o* (sometimes writing *r*_*t*_ for the reward following the *t* ^th^ action). From the utilities, we define the reward function *R*: 𝓐 × 𝓢 → ℝ, as the expected gain for taking action *a* at state *s* as *R*(*a*, *s*) = 𝔼[*r*(*a*, *s*, *o*)], where this expectation is taken over all possible observations *o*. Since we usually operate on histories, rather than fixed states, we define the expected reward from a given history *h* as *R*(*a*, *h*): = ∑_*s* ∈ 𝓢_ *R*(*a*, *s*)ℙ[*s*∣*h*]. The discount function weights the present impact of a future return, depending only on the separation between present and future. We use exponential discounting with a fixed number *γ* ∈ [0, 1] to define our discount function:

$$\begin{array}{c} \Gamma \left(\tau -t\right)=\gamma^{\tau -t}\;\forall \tau ,t\in \mathbb{N},\tau \geq t. \end{array}$$ (1)

Additionally, we define *H* such that *H*(*τ*, *t*) is 0 for *τ* > *K* and 1 otherwise. *K* in general is a random stopping time. We call the second component *t* the reference time of the survival function.

The survival function allows us to encode the planning horizon of an agent during decision making: If *H*(*τ*, *t*) is 0 for *τ*−*t* > *P*, we say that the local planning horizon at *t* is less than or equal to *P*.

The policy *π* ∈ Π, *π*(*a*, *h*): = ℙ[*a*∣*h*] is defined as a mapping of histories to probabilities over possible actions. Here Π is called the set of admissible policies. For convenience, we sometimes write the distribution function as *π*(*h*). The value function of a fixed policy *π* starting from present history *h*_*t*_ is

$$\begin{array}{c} V^{\pi }\left(h_{t}\right):=\sum_{\tau =t}^{\infty }\gamma^{\tau -t}H\left(\tau ,t\right)𝔼\left[r_{\tau }|\pi ,h_{\tau }\right] \end{array}$$ (2)

i.e., a sum of the discounted future expected rewards (note that *h*_*τ*_ is a random variable here, not a fixed value). Equally, the state-action value is

$$\begin{array}{c} Q^{\pi }\left(a,h_{t}\right):=R\left(a,h_{t}\right)+\sum_{\tau =t+1}^{\infty }\gamma^{\tau -t}H\left(\tau ,t\right)𝔼\left[r_{\tau }|\pi ,h_{\tau }\right]. \end{array}$$ (3)

**Definition 1** **(Formal Definition—POMDP).** Using the notation of this section, a POMDP is defined as a tuple (*𝓢, 𝓐, 𝓞, 𝓣, 𝓦, ℛ, Π, 𝓑_0_*) of components as outlined above.

**Convention 1** **(Softmax Decision Making).** A wealth of experimental work (for instance [27–29]) has found that the choices of humans (and other animals) can be well described by softmax policies based on the agent’s state-action values, to encompass the stochasticity of observed behaviour in real subject data. See [30], for a behavioural economics perspective and [10] for a neuroscience perspective. In view of using our model primarily for experimental analysis, we will base our discussion on the decision making rule:

$$\begin{array}{c} \pi \left(a,h\right)=\mathbb{P}\left[a|h\right]=\frac{e^{\beta Q^{\pi }\left(a,h\right)}}{\sum_{b\in 𝓐}e^{\beta Q^{\pi }\left(b,h\right)}} \end{array}$$ (4)

where β > 0 is called the inverse temperature parameter and controls how diffuse are the probabilities. The policy

$$\pi (a,h)=\{\begin{array}{ll} 1 & \text{if}Q^{\pi }(a,h)=\max\{Q^{\pi }(b,h)|b\in A\}\;(\text{assumingthisisunique})\\ 0 & \text{otherwise} \end{array}$$ (5)

can be obtained as a limiting case for β → ∞.

**Convention 2**. From now on, we shall denote by *Q*(*a*, *h*), the state-action value *Q* ^π^(*a*, *h*) with respect to the softmax policy.

### POMCP

POMCP was introduced by [31] as an efficient approximation scheme for solving POMDPs. Here, for completeness, we describe the algorithm; later, we adapt it to the case of an IPOMDP.

**Fig 3:**
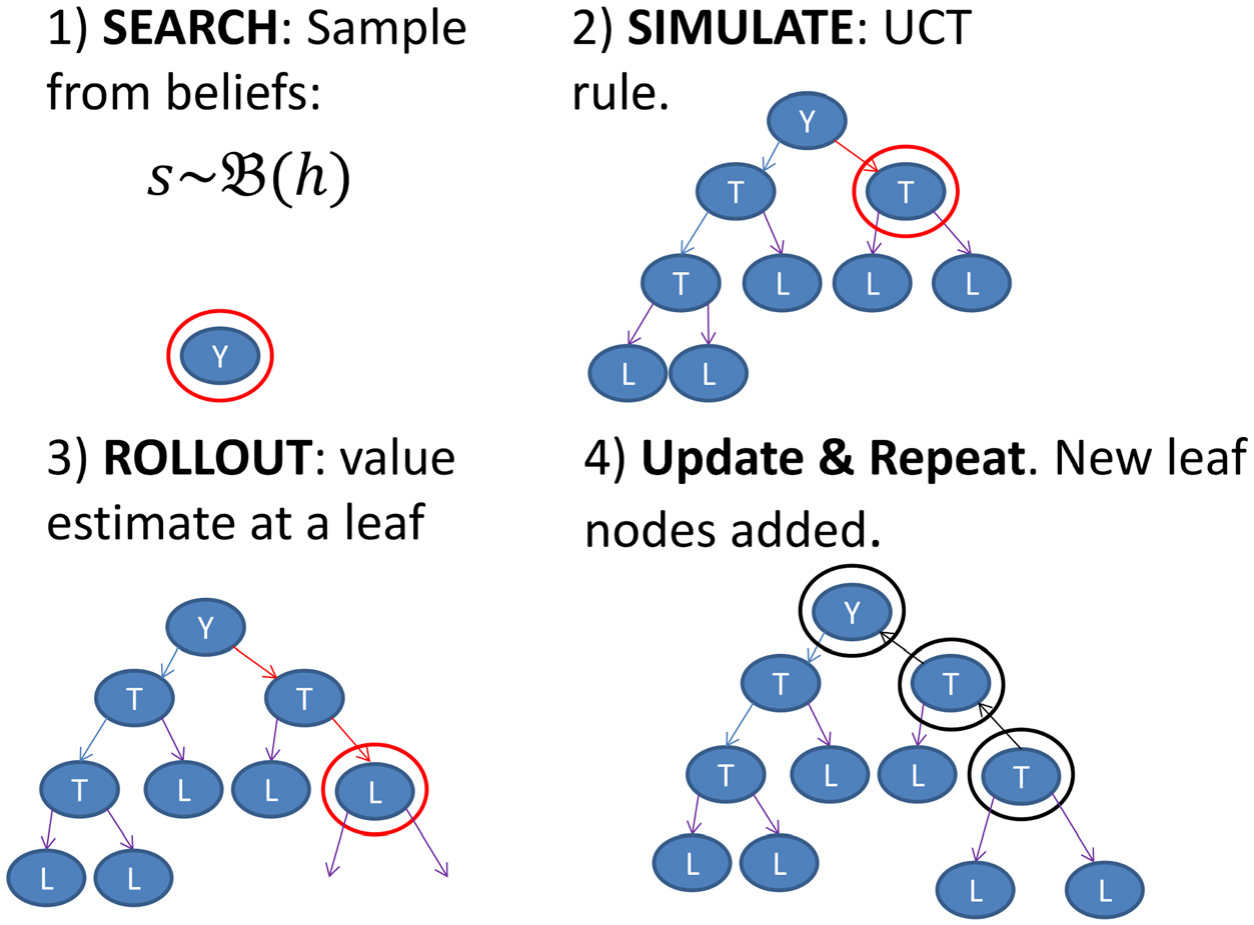
Illustration of POMCP. The algorithm samples a state *s* from the Belief state 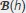 at the root Y (Y representing the current history *h*), keeps this state *s* fixed till step 4), follows UCT in already visited domains (labelled tree nodes *T*) and performs a rollout and Bellman backup when hitting a leaf (labelled *L*). Then step 1)–4) is repeated until the specified number of simulations has been reached.

POMCP is a generative model-based sampling method for calculating history-action values. That is, it builds a limited portion of the tree of future histories starting from the current *h*_*t*_, using a sample-based search algorithm (called upper confidence bounds for trees (UCT); [32]) which provides guarantees as to how far from optimal the resulting action can be, given a certain number of samples (based on results in [33] and [34]). Algorithm 1 provides pseudo code for the adapted POMCP algorithm. The procedure is presented schematically in Fig 3.

The algorithm is based on a tree structure *T*, wherein nodes $T(h)=(N(h),\tilde{Q}(h),𝓑(h))$ represent possible future histories explored by the algorithm, and are characterized by the number *N*(*h*) of times history *h* was visited in the simulation, the estimated value $\tilde{Q}(h)$ for visiting *h* and the approximate belief state 𝓑(*h*) at *h*. Each new node in *T* is initialized with initial action exploration counts *N*(*h*, *a*) = 0 for all possible actions *a* from *h* and an initial action value estimate $\tilde{Q}(h,a)=0$ for all possible actions *a* from *h* and an empty belief state 𝓑(*h*) = ∅.

The value *N*(*h*) is then calculated from all actions counts from the node *N*(*h*) = ∑_*a* ∈ 𝓐_ *N*(*h*, *a*). $\tilde{Q}(h)$ denotes the mean of obtained values, for simulations starting from node *h*. 𝓑(*h*) can either be calculated analytically, if it is computationally feasible to apply Bayes theorem, or be approximated by the so called *root sampling* procedure (see below).

In terms of the algorithm, the generative model 𝓖 of the POMDP determines (*s* ^′^, *o*, *r*) ∼ 𝓖(*s*, *a*), the simulated reward, observation and subsequent state for taking *a* at *s*; *s* itself is sampled from the current history *h*. Then, every (future) history of actions and observations *h* defines a node *T*(*h*) in the tree structure *T*, which is characterized by the available actions and their average simulated action values $\tilde{Q}(a,h)$ under the policy SoftUCT at future states.

**Algorithm 1:** Partially Observable Monte Carlo Planning.

**procedure** SEARCH(h, t, n)	**procedure** SIMULATE (s, h, t, k)
**for** SIMULATIONS = 1, …, *n* **do**	**if** *H*(*k*, *t*) ≤ 0 **then**
*k* ← *t*	**return** 0
**if** *h_t_* = *o*_0_ **then**	**end if**
*s* ∼ *𝓑*_0_	**if** *h* ∉ T **then**
**else**	**for all** *a* ∈ *𝓐* do
*s* ∼ *𝓑*(*h_t_*)	*T*(*ha*) ← (*N*(*h*, *a*); *Q*(*a*, *h*), ∅)
**end if**	
SIMULATE (*s, h, t, k*)	**end for**
**end for**	**return** ROLLOUT (*s, h, t, k*)
**return** *a* ∼ SoftUCT(Q(.|h))	**end if**
**end procedure**	
**procedure** ROLLOUT(*s, h, t, k*)	*a* ∼ SoftUCT(Q(.|h))
**if** *H*(*k*, *t*) ≤ 0 **then**	(*s*′, *o*, *r*) ∼ *𝓖*(*s*, *a*)
**return** 0	*h* ← {*h*, *a*, *o*}
**end if**	*k* ← *k* + 1
*a* ∼ π_rollout_(*h*, ·)	*R* ← *r*+*γ*SIMULATE(*s′, h, t, k*)
(*s*′, *o*, *r*) ∼ 𝓖(*s*, *a*)	*N(h)* ← *N(h)* + 1
*h* ← {*h, a, o*}	*N(h, a)* ← *N(h, a)* + 1
*k* ← *k* + 1	$\tilde{Q}(a,h)\leftarrow \tilde{Q}(a,h)+\frac{R-\tilde{Q}(a,h)}{N(h,a)}$
**return** *r*+*γ*ROLLOUT(*s′ h, t, k*)	**return R**
**end procedure**	**end procedure**

If the node has been visited for the *N*(*h*)^th^ time; with action *a* being taken for the *N*(*h*, *a*)^th^ time, then the average simulated value is updated (starting from 0) using sampled simulated rewards *R* up to terminal time *K*, when the current simulation/tree traversal ends as:

$$\begin{array}{c} {\tilde{Q}}^{\mathrm{new}}\left(a,h\right)={\tilde{Q}}^{\mathrm{old}}\left(a,h\right)+\frac{1}{N(h,a)}(R-{\tilde{Q}}^{\mathrm{old}}\left(a,h\right)). \end{array}$$ (6)

The search algorithm has two decision rules, depending on whether a traversed node has already been visited or is a leaf of the search tree. In the former case, a decision is reached using SoftUCT by defining

$$\begin{array}{c} \text{SoftUCT(Q(.}|\text{h))}\;Q\left(a,h\right):=\tilde{Q}\left(a,h\right)+c\sqrt{\frac{\log N(h)}{N(h,a)}}\;\mathbb{P}\left[a|h\right]=\frac{e^{\beta (Q(a,h))}}{\sum_{b}e^{\beta (Q(b,h))}} \end{array}$$ (7)

where *c* is a parameter that favors exploration (analogous to an equivalent parameter in UCT).

If the node is new, a so-called “rollout” policy is used to provide a crude estimate of the value of the leaf. This policy can be either very simple (uniform or *ε*–greedy based on a very simple model) or specifically adjusted to the search space, in order to optimize performance.

The rollout value estimate together with the SoftUCT exploration rule is the core mechanism for efficient tree exploration. In this work, we only use an *ε*–greedy mechanism, as is described in the section on the multi round trust game.

Another innovation in POMCP that underlies its dramatically superior performance is called *root sampling*. This procedure allows to form the belief state at later states, as long as the initial belief state 𝓑_0_ is known. This means that, although it is necessary to perform inference to draw samples from the belief state at the root of the search tree, one can then use each sample as if it was (temporarily) true, without performing inference at states that are deeper in the search tree to work out the new transition probabilities that pertain to the new belief states associated with the histories at those points. The reason for this is that the probabilities of getting to the nodes in the search tree represent exactly what is necessary to compensate for the apparent inferential infelicity [31]– i.e., the search tree performs as a probabilistic filter. The technical details of the root sampling procedure can be found in [31].

In the presence of analytically tractable updating rules (or at least analytically tractable approximations), the belief state at a new node can instead be calculated by Bayes’ theorem. This will also be the case for the multi round trust game below, where we follow the approximate updating rule in [22].

### Interactive Partially Observable Markov Decision Processes

An Interactive Partially Observable Markov Decision Process (IPOMDP) is a multi agent setting in which the actions of each agent may observably affect the distribution of expected rewards for the other agents.

Since IPOMDPs may be less familiar than POMDPs, we provide more detail about them; consult [23] for a complete reference formulation and [35] for an excellent discussion and extension.

We define the IPOMDP such that the decision making process of each agent becomes a standard (albeit large) POMDP, allowing the direct application of POMDP methods to IPOMDP problems.

**Fig 4:**
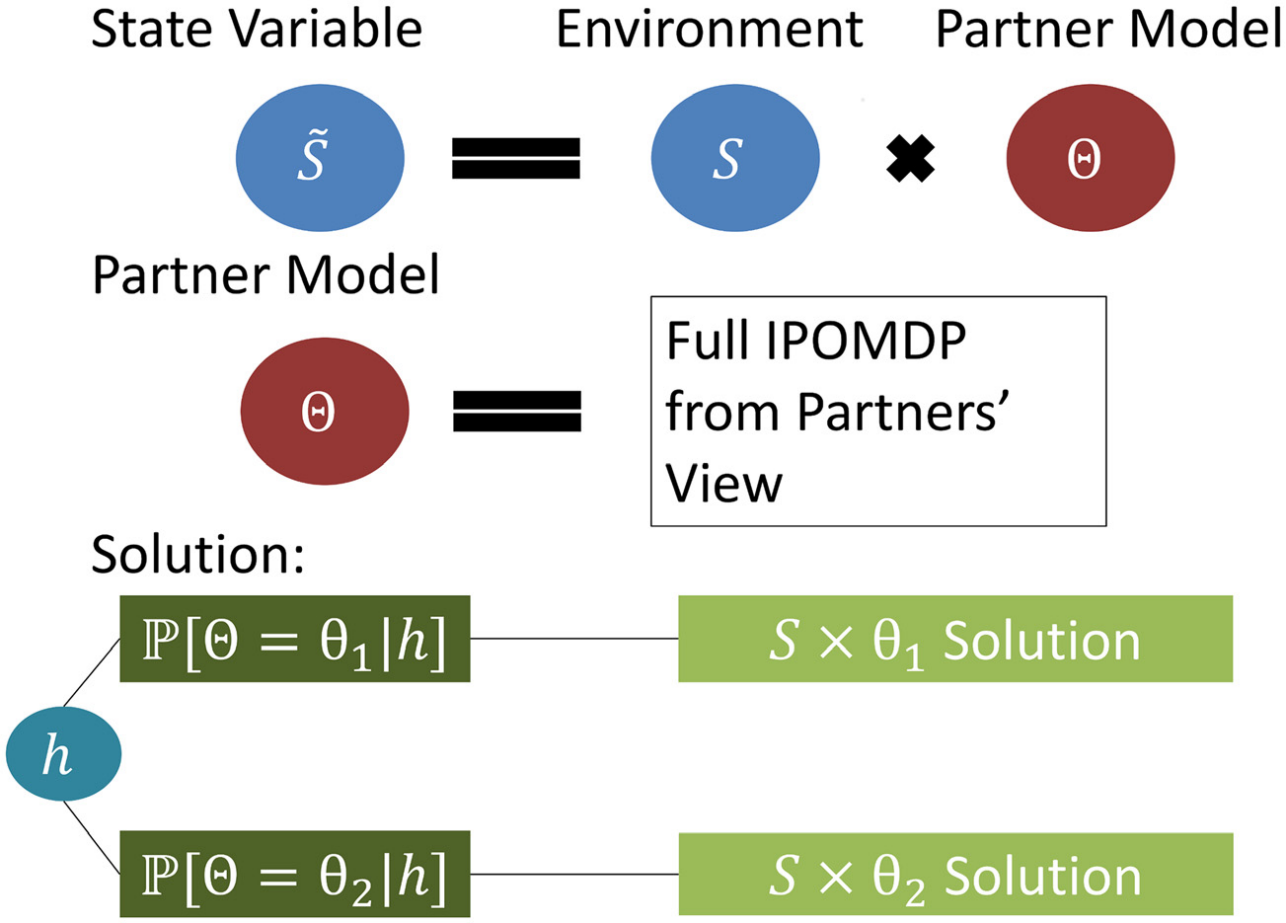
Interactive partially observable Markov decision process. Compared to a POMDP, the process is further complicated by the necessity to keep different models Θ of the other agent’s intentions, so that evidence about the correct intentional model may be accrued in the belief state 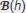. The IPOMDP solution requires to integrate over all possible states and intentional models according to the belief state at every possible history.

**Definition 2 (Formal Definition—IPOMDP).** An IPOMDP is a collection of POMDPs such that the following holds:

Agents are indexed by the finite set ℐ. Each agent i ∈ ℐ is described by a single POMDP (𝓢^i^, 𝓐^i^, 𝓞^i^, 𝓣^i^, 𝓦^i^, ℛ^i^, Π^i^, ${𝓑}_{0}^{i})$ denoting its actual decision making process. We first define the physical state space ${𝓢}_{\text{phys}}^{i}$: an element ${𝓢}^{i}\in {𝓢}_{\text{phys}}^{i}$ is a complete setting of all features of the environment that determine the action possibilities 𝓐^i^ and obtainable rewards ℛ^i^ of i for the present and all possible following histories, from the point of view of i. The physical state space ${𝓢}_{\text{phys}}^{i}$ is augmented by the set 𝓓^i^ of models of the partner agents θ^ij^ ∈ 𝓓^i^, j ∈ ℐ\{i}, called intentional models, which are themselves POMDPs θ^ij^ = (𝓢^ij^, 𝓐^ij^, 𝓞^ij^, 𝓣^ij^, 𝓦^ij^, ℛ^ij^, Π^ij^, ${𝓑}_{0}^{ij})$. These describe how agent i believes agent j perceives the world and reaches its decisions. The possible state space of agent i can be written ${𝓢}^{i}={𝓢}_{\text{phys}}^{i}\times {𝓓}^{i}$ and a given state can be written ${\tilde{s}}^{i}=(s^{i},\times_{j}\theta^{ij})$, where $s^{i}\in {𝓢}_{\text{phys}}^{i}$ is the physical state of the environment and θ^ij^ are the models of the other agents. Note that the intentional models θ^ij^ contain themselves state spaces that encode the history of the game as observed by agent j from the point of view of agent i. The elements of 𝓢^i^ are called interactive states. Agents themselves act according to the softmax function of history-action values, and assume that their interactive partner agents do the same. The elements of the definition are summarized in Fig 4.

**Convention 3**. We denote by *S* and $\tilde{S}$ the random variables, that encode uncertainty about the physical state and the interactive state respectively.

**Fig 5:**
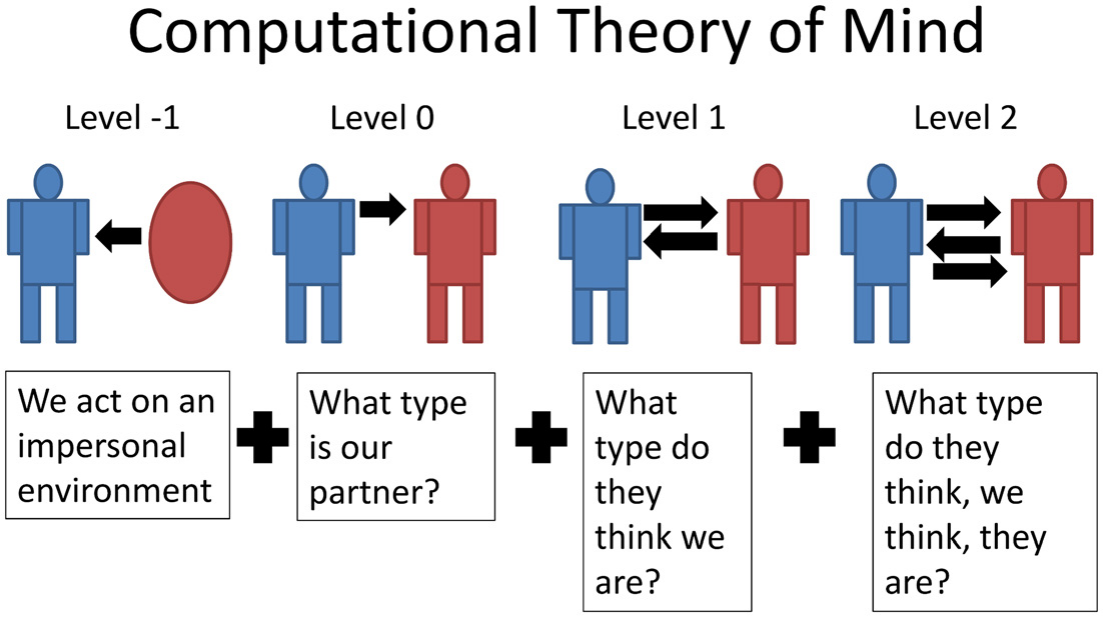
Computational theory of mind (ToM) formalizes the notion of our understanding of other peoples’ thought processes.

When choosing the set of intentional models, we consider agents and their partners to engage in a cognitive hierarchy of successive mentalization steps [9, 12], depicted in Fig 5. The simplest agent can try to infer what kind of partner it faces (level 0 thinking). The next simplest agent could additionally try to infer what the partner might be thinking of it (level 1). Next, the agent might try to understand their partner’s inferences about the agent’s thinking about the partner (level 2). Generally, this would enable a potentially unbounded chain of mentalization steps. It is a tenet of cognitive hierarchy theory [9] that the hierarchy terminates finitely and for many tasks after only very few steps (e.g., Poisson, with a mean of around 1.5).

We formalize this notion as follows.

**Definition 3 (A Hierarchy Of Intentional Models).** Since models of the partner agent may contain interactive states in which it in turn models the agent i, we can specify a hierarchical intentional structure 𝓓^i, l^, built from what we call the level l ≥ −1 intentional models 𝓓^i,l^. 𝓓^i,l^ is defined inductively from

$$\begin{array}{c} \theta^{ij,-1}\in {𝓓}^{i,-1}\Leftrightarrow S^{ij,-1}={𝓢}_{\mathrm{phys}}^{ij}\times \left\{\emptyset \right\}. \end{array}$$ (8)

This means that any level −1 intentional model reacts strictly to the environment, without holding any further intentional models. The higher levels are obtained as

$$\begin{array}{c} \theta^{ij,l}\in {𝓓}^{i,l}\Leftrightarrow {𝓢}^{ij,l}={𝓢}_{\mathrm{phys}}^{ij}\times {𝓓}^{ij,l-1}. \end{array}$$ (9)

Here 𝓓^ij, l−1^ denotes the l−1 intentional models, that agent i thinks agent j might hold of the other players. These level l−1 intentional models arise by the same procedure applied to the level −1 models that agent i thinks agent j might hold.

**Definition 4 (Theory of Mind (ToM) Level).** We follow a similar assumption as the so called k-level thinking (see [12]), in that we assume that each agent operates at a particular level l^i^ (called the agent’s theory of mind (ToM) level; and which it is assumed to know), and models all partners as being at level l^j^ = l^i^−1.

We chose definition 4 for comparability with earlier work [22, 24].

**Convention 4.** It is necessary to be able to calculate the belief state in every POMDP that is encountered. An agent updates its belief state in a Bayesian manner, following an action $a_{t}^{i}$ and an observation $o_{t+1}^{i}$. This leads to a sequential update rule operating over the belief state $\mathbb{P}[{\tilde{S}}_{t}^{i}|h_{t}^{i}]$ of a given agent i at a given time t:

$$\begin{array}{c} \mathbb{P}\left[{\tilde{S}}_{t+1}^{i}={\tilde{s}}_{1}|\left\{h_{t}^{i},a_{t}^{i},o_{t+1}^{i}\right\}\right]=\eta 𝓦\left(o_{t+1}^{i},a_{t}^{i},{\tilde{s}}_{1}\right)\sum_{\tilde{s}\in {𝓢}^{i}}𝓣\left({\tilde{s}}_{1},a_{t}^{i},\tilde{s}\right)\mathbb{P}\left[{\tilde{S}}_{t}^{i}=\tilde{s}|h_{t}^{i}\right]. \end{array}$$ (10)

Here η is a normalization constant associated with the joint distribution of transition and observation probability, conditional on $\tilde{s}$, ${\tilde{s}}_{1},o_{t+1}^{i}$ and $a_{t}^{i}$. The observation $o_{t+1}^{i}$ in particular incorporates any results of the actions of the other agents, before the next action of the given agent.

We note that the above rule applies recursively to every intentional model in the nested structure 𝓓^i^, as every POMDP has a separate belief state.

This is slightly different from [23] so that the above update is conventional for a POMDP.

**Convention 5.** (Expected Utility Maximisation). The decision making rule in our IPOMDP treatment is based on expected utility as encoded in the reward function. The explicit formula for the action value $Q(a_{t}^{i},h_{t}^{i})$ under a softmax policy (Eq (4)) is:

$$\begin{array}{c} Q\left(a_{t}^{i},h_{t}^{i}\right)=R\left(a_{t}^{i},h_{t}^{i}\right)+\sum_{o_{t+1}^{i}\in 𝓞}\mathbb{P}\left[o_{t+1}^{i}|\left\{h_{t}^{i},a_{t}^{i}\right\}\right]\sum_{w\in {𝓐}^{i}}\gamma^{\left(i\right)}H\left(t+1,t\right)Q\left(w,h_{t+1}^{i}|t\right)\mathbb{P}\left[b|h_{t+1}^{i}\right]. \end{array}$$ (11)

Here $h_{t+1}=\{h_{t}^{i},a_{t}^{i},o_{t+1}^{i}\}$ and $Q(b,h_{t+1}^{i}|t)$ denotes the action value at t+1 with the survival function conditioned to reference time t. γ^(i)^ is the discount factor of agent i, rather than the i-th power. This defines a recursive Bellman equation, with the value of taking action $a_{t}^{i}$ given history $h_{t}^{i}$ being the expected immediate reward $R(a_{t}^{i},h_{t}^{i})$ plus the expected value of future actions conditional on $a_{t}^{i}$ and its possible consequences $o_{t+1}^{i}$ discounted by γ^i^.

The belief state $𝓑(h_{t}^{i})$ allows us to link $h_{t}^{i}$ to a distribution of interactive states and use 𝓦 to calculate $\mathbb{P}[o_{t+1}^{i}|\{h_{t}^{i},a_{t}^{i}\}]$ , in particular including the reactions of other agents to the actions of one agent. We call the resulting policy the “solution” to the IPOMDP.

### Equilibria and IPOMDPs

Our central interest is in the use of the IPOMDP to capture the interaction amongst human agents with limited cognitive resources and time for their exchanges. It has been noted in [9] that the distribution of subject levels favours rather low values (e.g., Poisson, with a mean of around 1.5). In the opposite limit, sufficient conditions are known in which taking the cognitive hierarchy out to infinity for all involved agents allows for at least one Bayes-Nash equilibrium solution (part II, theorem II, p. 322 of Harsanyi [8]) and sufficient conditions have been shown in [36], given which a solution to the infinite hierarchy model can be approximated by the sequence of finite hierarchy model solutions. A discussion of a different condition can be found in [37]; however, this condition does assume a infinite time horizon in the interaction. In general, as [9], p.868 notes, it is not true that the infinite hierarchy solution will be a Nash equilibrium. For the purposes of computational psychiatry, we find the very mismatches and limitations, that prevent subjects’ strategies to evolve to a (Bayes)-Nash equilibrium in the given time frame, to be of particular interest. Therefore we restrict our attention to quantal response equilibrium like behaviours ([30]), based on potentially inconsistent initial beliefs by the involved agents with ultimately very limited cognitive resources and finite time exchanges.

### Applying POMCP to an IPOMDP

An IPOMDP is a collection of POMDPs, so POMCP is, in principle, applicable to each encountered POMDP.

However, unlike the examples in [31], an IPOMDP contains the intentional model POMDPs *θ* ^*ij*^ as part of the state space, and these themselves contain a rich structure of beliefs. So, the state is sampled from the belief state at the root for agent *i* is an *I* tuple $\left({\hat{s}}^{i},{\hat{\theta }}^{i1},\ldots ,{\hat{\theta }}^{i(|ℐ|-1)}\right)$ of a physical state ${\hat{s}}^{i}$ and (∣ℐ∣−1) POMDPs, one for each partner. (This is also akin to the random instantiation of players in [8]). Since the ${\hat{\theta }}^{ij}$ still contain belief states in their own right, it is still necessary to do some explicit inference during the creation of each tree. Indeed, explicit inference is hard to avoid altogether during simulation, as the interactive states require the partner to be able to learn [23]. Nevertheless, a number of performance improvements that we detail below still allow us to apply the POMCP method involving substantial planning horizons.

### Simplifications for Dydadic Repeated Exchange

Many social paradigms based upon game theory, including the iterated ultimatum game, prisoners’ dilemma, iterated “rock, paper, scissors” (for 2 agents) and the multi round trust game, involve repeated dyads. In these, each interaction involves the same structure of physical states and actions (𝓢_phys_, 𝓐) (see below), and all discount functions are 0 past a finite horizon.

**Definition 5 (Dyadic Repeated Exchange without state uncertainty).** Consider a two agent IPOMDP framework in which there is no physical state uncertainty: both agents fully observe each others’ actions and there is no uncertainty about environmental influence; and in which agents vary their play only based on intentional models. Additionally, the framework is assumed to reset after each exchange (i.e., after both agents have acted once).

Formally this means: There is a fixed setting (𝓢_phys_, 𝓐, 𝓣), such that physical states, actions from these states, transitions in the physical state and hence also obtainable rewards, differ only by a changing time index and there is no observational uncertainty. Then after each exchange the framework is assumed to reset to the same distribution of physical initial states 𝓢_phys_ within this setting (i.e. the game begins anew).

Games of this sort admit an immediate simplification:

**Theorem 1 (Level 0 Recombining Tree).** In the situation of definition 5, level 0 action values at any given time only depend on the total set of actions and observations so far and not the order in which those exchanges were observed.

*Proof*. The level −1 partner model only acts on the physical state it encounters and the physical state space variable *S* is reset at the beginning of each round in the situation of 5. Therefore, given a state *s* in the current round and an action *a* by a level 0 agent, the likelihood of each transition to some state *s*_1_, 𝓣(*s*_1_, *a*, *s*), and of making observation *o*, 𝓦(*o*, *a*, *s*_1_), is the same at every round from the point of view of the level 0 agent. It follows that the cumulative belief update from Eq 10, from the initial beliefs 𝓑_0_ to the current beliefs, will not depend on the order in which the action observation pairs (*a*, *o*) were observed.

This means, that depending on the size of the state space and the depth of planning of interest, we may analytically calculate level 0 action values even online or use precalculated values for larger problems. Furthermore, because their action values will only depend on past exchanges and not on the order in which they were observed, their decision making tree can be reformulated as a recombining tree.

Sometimes, an additional simplification can be made:

**Theorem 2 (Trivialised Planning).** In the situation of definition 5, if the two agents do not act simultaneously and the state transition of the second agent is entirely dependent on the action executed by the first agent (as in the multi round trust task); and additionally the intentional model of the partner can not be changed through the actions of the second agent, then a level 0 second agent can gain no advantage from planning ahead, since their actions will not change the action choices of the first agent.

*Proof*. In the scenario described in theorem 2 the physical state variable *S* of the agent 2 is entirely dependent on the action of the other agent. If the agent is level 0, they model their partner as level −1 and by additional assumption the second agent does not believe that the partner can be made to transition between different intentional models by the second agent’s actions, hence their partner will not change their distribution of state transitions, depending on the agent’s actions and hence also their distribution of future obtainable rewards will not change.

**Theorem 3 (Trivialised Theory of Mind Levels).** In the situation of theorem 2, we state that for the first to go agent, only the even theory of mind levels *k* ∈ {0}∪2ℕ show distinct behaviours, while the odd levels *k* ∈ 2ℕ−1 behave like one level below, meaning *k*−1. For the second to go partner equivalently, only the odd levels *k* ∈ {0}∪2ℕ−1 show distinct behaviours.

*Proof*. In the scenario described in theorem 2, the second to go level 0 agent behaves like a level −1 agent, as it does not benefit from modeling the partner. This implies that the first to go agent, gains no additional information at the level 1 thinking, since the partner behaves like level −1, which was modeled by the level 0 first to agent already. In turn, the level 2 second to go agent gains no additional information over the level 1 second to go agent, as the their partner model does not change between modeling the partner at level 0 or level −1. By induction, we get the result.

Examples of the additional simplifications in theorems 2 and 3 can be seen in the ultimatum game and the multi round trust game.

### The Trust Task

**Fig 6:**
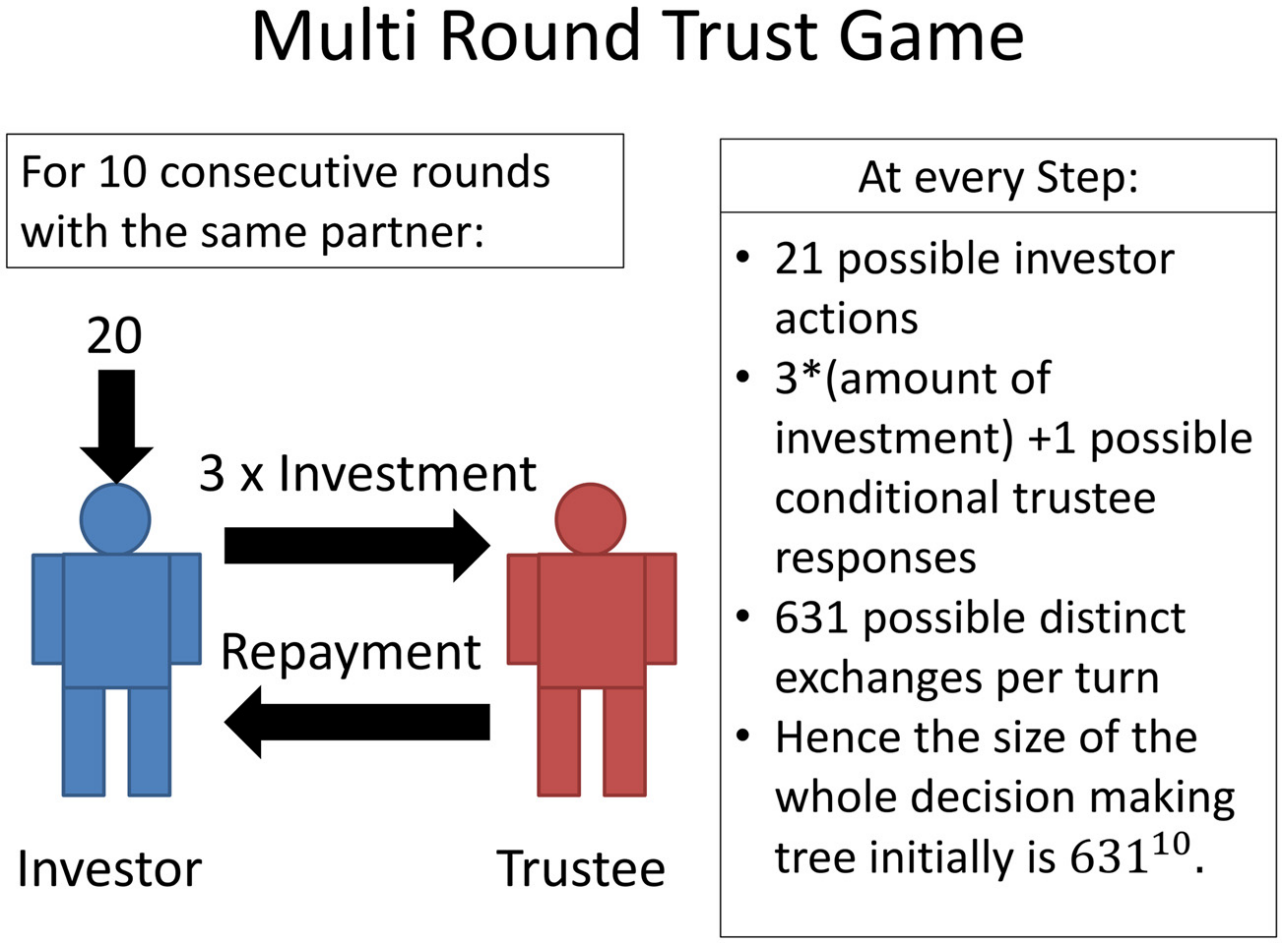
Physical features of the multi round trust game.

The multi-round trust task, illustrated in Fig 6 is a paradigm social exchange game. It involves two people, one playing the role of an ‘investor’ the other the one of a ‘trustee’, over 10 sequential rounds, expressed by a time index *t* = 1,2,…,10.

**Fig 7:**
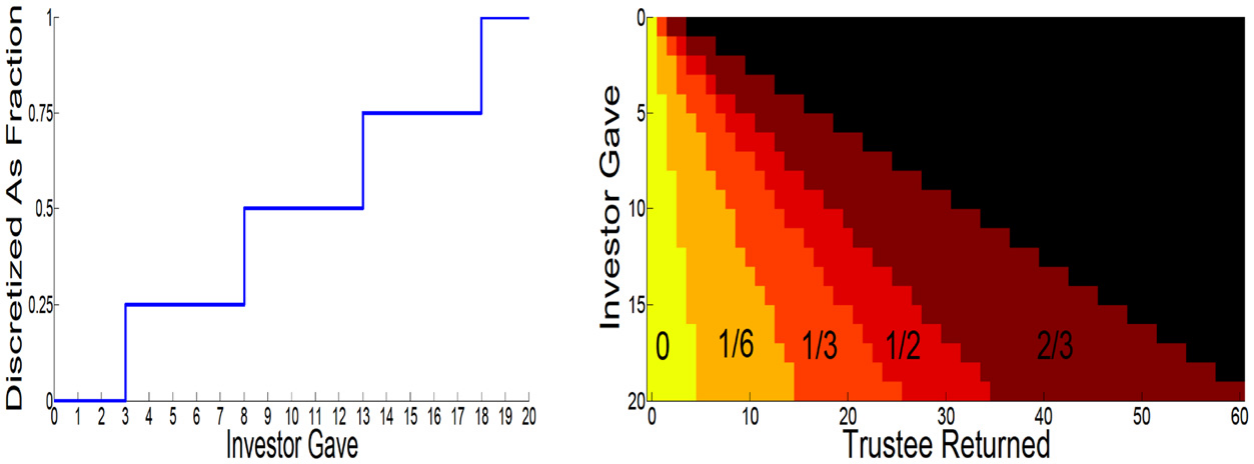
Discretized actions of both players. Investor: (left) The 21 possible actions are summarized into 5 possible investment categories. Trustee: (right) returns are classified into 5 possible categories, conditionally on investor action. Impossible returns are marked in black.

Both agents know all the rules of the game. In each round, the investor receives an initial endowment of 20 monetary units. The investor can send any of this amount to the trustee. The experimenter trebles this quantity and then the trustee decides how much to send back to the investor, between 0 points and the whole amount that she receives. The repayment by the trustee is not increased by the experimenter. After the trustee’s action, the investor is informed, and the next round starts. We consider the trust task as an IPOMDP with two agents, i.e., ℐ = {*I*, *T*} contains just *I* for the investor and *T* for the trustee. We consider the state to contain two components; one physical and observable (the endowment and investments), the other non-physical and non-observable (in our case, parameters of the utility function). It is the latter that leads to the partial observability in the IPOMDP. Following [24], we reduce complexity by quantizing the actions and the (non-observable) states of both investor and trustee—shown for one complete round in Fig 7.

The actions are quantized into 5 fractional categories shown in Fig 7. For the investor, we consider *a* ^*I*^ ∈ {0,0.25,0.5,0.75,1} (corresponding to an investment of $20 × *a* ^*I*^, and encompassing even investment ranges). For the trustee, we consider *a* ^*T*^ ∈ {0,0.167,0.333,0.5,0.67} (corresponding to a return of $3 × 20 × *a* ^*I*^ × *a* ^*T*^, and encompassing even return ranges). Note that the trustee’s action is degenerate if the investor gives 0. The pure monetary payoffs for both agents in each round are

$$\begin{array}{c} \mathrm{investor}:\chi^{I}\left(a^{I},a^{T}\right)=20-20\times a^{I}+3\times 20a^{I}\times a^{T} \end{array}$$ (12)

$$\begin{array}{c} \mathrm{trustee}:\chi^{T}\left(a^{I},a^{T}\right)=3\times 20\times a^{I}-3\times 20a^{I}\times a^{T}. \end{array}$$ (13)

**Fig 8:**
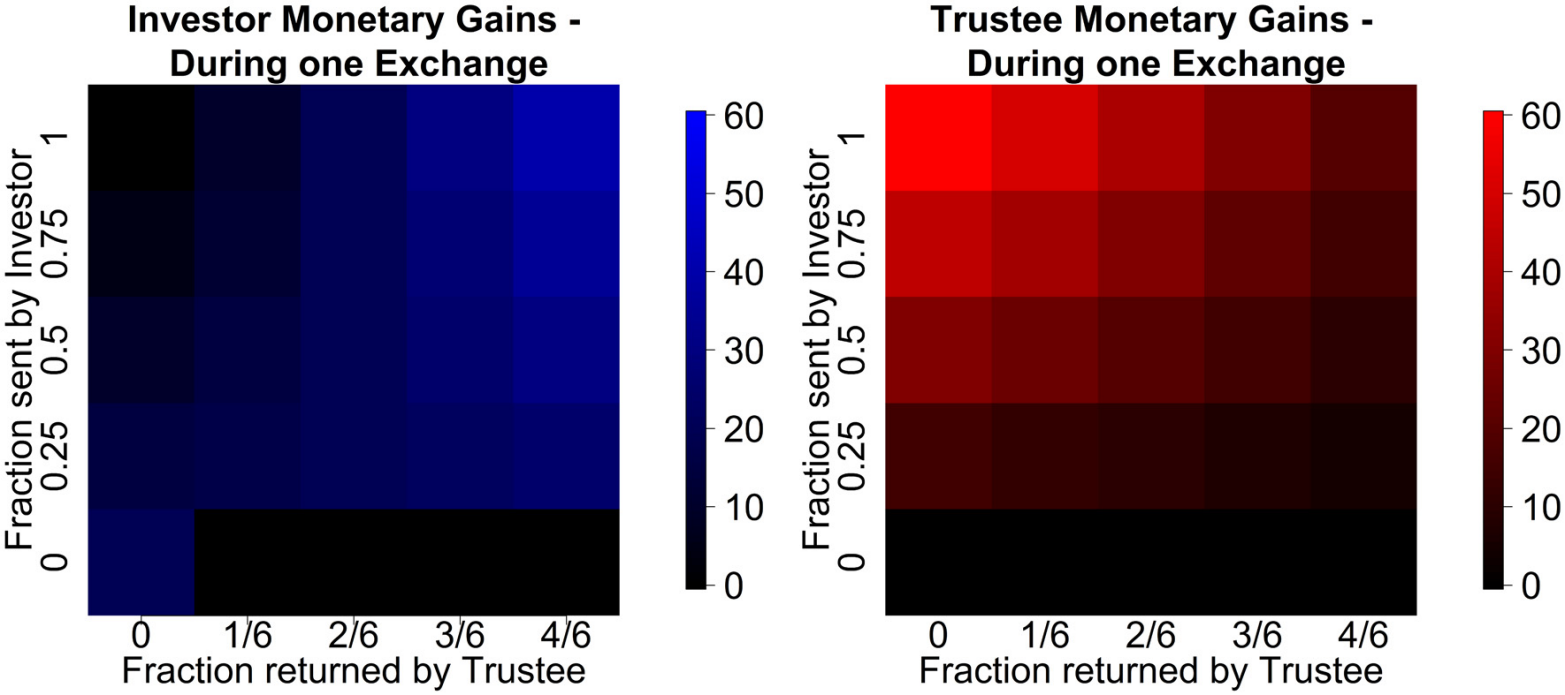
Payoffs in the multi round trust task. (left) Investor payoffs for an single exchange. (right) Trustee payoffs for an single exchange.

The payoffs of all possible combinations and both partners are depicted in Fig 8.

**Fig 9:**
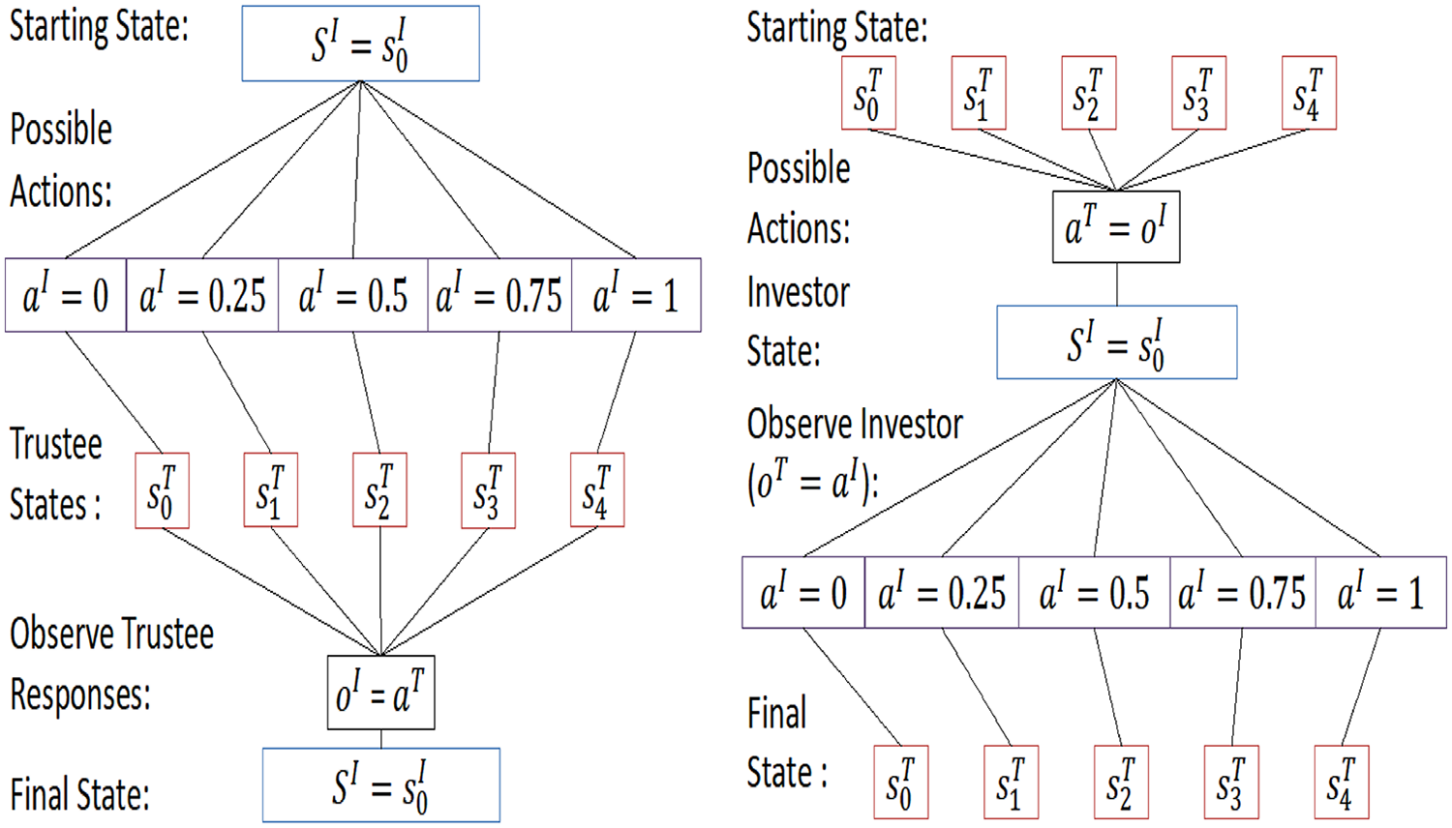
(Physical) transitions and observations: (Left) physical state transitions and observations of the investor. The trustee’s actions are summarized to *a* ^*T*^, as they can not change the following physical state transition. (right) Physical state transitions and observations of the trustee. The trustee’s actions are summarized to *a* ^*T*^, as they can not change the following physical state transition.

In IPOMDP terms, the investor’s physical state is static, whereas the trustee’s state space is conditional on the previous action of the investor. The investor’s possible observations are the trustees responses, with a likelihood that depends entirely on the investor’s intentional model of the trustee. The trustee observes the investor’s action, which also determines the trustee’s new physical state, as shown in Fig 9.

#### Inequality aversion—compulsion to fairness

The aspects of the states of investor and trustee that induce partial observability are assumed to arise from differential levels of cooperation.

**Fig 10:**
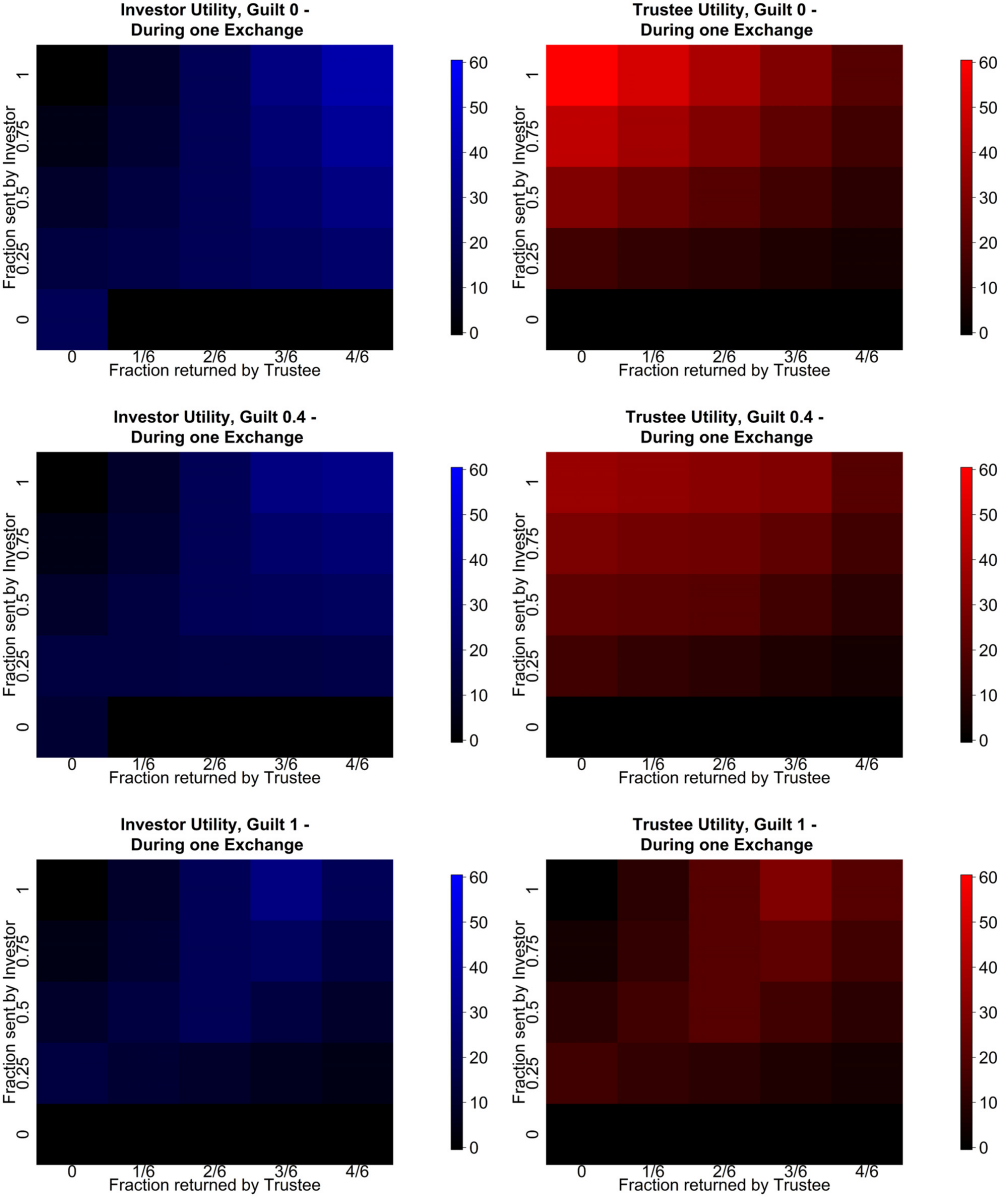
Immediate Fehr-Schmidt utilities for a single exchange [1]. Left column shows investor preferences: (top left) Completely unguilty investor values only the immediate payoff, (middle left) Guilt 0.4 investor is less likely to keep everything to themselves (bottom left corner option), (bottom left) Guilt 1 investor will never keep everything to themselves (bottom left option). Right column shows trustee preferences: (top right) unguilty trusty would like to keep everything to themselves. (middle right) Guilt 0.4 is more likely to return at least a fraction of the gains. (bottom right) Guilt 1 trustee will strife to return the fair split always.

One convenient (though not unique) way to characterize this is via the Fehr-Schmidt inequality aversion utility function (Fig 10). This allows us to account for the observation that many trustees return an even split even on the last exchange of the 10 rounds, even though no further gain is possible.

We make no claim that this is the only explanation for such behaviour, but it is a tractable and well-established mechanism that has been used successfully in other tasks ([1, 14, 27]). For the investor, this suggests that:

$$\begin{array}{c} r^{I}\left(a^{I},a^{T},\alpha^{I}\right)=\chi^{I}\left(a^{I},a^{T}\right)-\alpha^{I}\;\max\left\{\chi^{I}\left(a^{I},a^{T}\right)-\chi^{T}\left(a^{I},a^{T}\right),0\right\}. \end{array}$$ (14)

Here, *α* ^*I*^ is called the “guilt” parameter of the investor and quantifies their aversion to unequal outcomes in their favor. We quantize guilt into 3 concrete guilt types {0,0.4,1} = {*α*_1_, *α*_2_, *α*_3_}. Similarly, the trustee’s utility is

$$\begin{array}{c} r^{T}\left(a^{I},a^{T},\alpha^{T}\right)=\chi^{T}\left(a^{I},a^{T}\right)-\alpha^{T}\;\max\left\{\chi^{T}\left(a^{I},a^{T}\right)-\chi^{I}\left(a^{I},a^{T}\right),0\right\} \end{array}$$ (15)

with the same possible guilt types. We choose these particular values, as guilt values above 0.5 tend to produce similar behaviours as *α* = 1 and the values below 0.3 tend to behave very similar to *α* = 0. Thus we take *α*_1_ to represent guilt values in [0,0.3], *α*_2_ to represent guilt values in (0.3,0.5) and *α*_2_ to represent guilt values in [0.5,1]. We assume that neither agent’s actual guilt type changes during the 10 exchanges.

#### Planning behaviour

The survival functions *H* ^*I*^ and *H* ^*T*^ are used to delimit the planning horizon. The agents are required not to plan beyond the end of the game at time 10 and within that constraint they are supposed to plan *P* steps ahead into the interaction. This results in the following form for the survival functions (regardless whether for investor or trustee):

$$\begin{array}{c} H_{P}\left(\tau ,t\right)=1\;\left(\tau -t\right)\leq P\wedge \left(\tau +t\right)\leq 10,\;H_{P}\left(\tau ,t\right)=0\;\left(\tau -t\right)>P\vee \left(\tau +t\right)>10. \end{array}$$ (16)

The value *P* is called the planning horizon. We consider *P* ∈ {0,2,7} for immediate, medium and long planning types. We chose these values as *P* = 7 covers the range of behaviours from *P* = 4 to *P* = 9, while planning 2 yields compatibility to earlier works ([22, 24]) and allows to have short planning but high level agents, covering the range of behaviours for planning *P* = 1 to *P* = 3. We confirm later that the behaviour of *P* = 7 and *P* = 9 agents is almost identical; and the former saves memory and processing time. Agents are characterized as assuming their opponents have the same degree of planning as they do. The discount factors *γ* ^*I*^ and *γ* ^*T*^ are set to 1 in our setting.

### Belief State

Since all agents use their own planning horizon in modeling the partner and level *k* agents model their partner at level *k*−1, inference in intentional models in this analysis is restricted to the guilt parameter *α*. Using a categorical distribution on the guilt parameter and Dirichlet prior on the probabilities of the categorical distribution, we get a Dirichlet-Multinomial distribution for the probabilities of an agent having a given guilt type at some point during the exchange. Hence 𝓑_0_ is a Dirichlet-Multinomial distribution,

$$\begin{array}{c} {𝓑}_{0}∼DirMult\left(a_{0}\right)\;a_{0}=\left(1,1,1\right) \end{array}$$ (17)

with the initial belief state

$$\begin{array}{c} \mathbb{P}\left[\alpha^{\mathrm{partner}}=\alpha_{i}|h=\emptyset \right]=\frac{1}{3}. \end{array}$$ (18)

Keeping consistent with the model in [22], our approximation of the posterior distribution is a Dirichlet-Multinomial distribution with the parameters of the Dirichlet prior being updated to

$$\begin{array}{c} a_{t+1}^{i}=a_{t}^{i}+\mathbb{P}\left[o_{t+1}=\mathrm{observed}\mathrm{action}|\alpha^{\mathrm{partner}}=\alpha_{i}\right] \end{array}$$ (19)

writing *α* ^partner^ for the intentional models.

#### Theory of mind levels and agent characterization

Since the physical state transition of the trustee is fully dependent on the investor’s action and one agent’s guilt type can not be changed by the actions of the other agent, theorem 2 implies that the level 0 trustee is trivial, gaining nothing from planning ahead. Conversely, the level 0 investor can use a recombining tree as in theorem 1. Therefore, the chain of cognitive hierarchy steps for the investor is *l* ^*I*^ ∈ {0}∪{2*n*∣*n* ∈ ℕ}, and for the trustee, it is *l* ^*T*^ ∈ {0}∪{2*n*−1∣*n* ∈ ℕ}. Trustee planning is trivial until the trustee does at least reach theory of mind level 1. Assuming $\beta =\frac{1}{3}$ in Eq 4, determined empirically from real subject data [22] for suitably noisy behaviour, our subjects are then characterized via the triplet (*k*, *α*, *P*) of theory of mind level *k*, guilt parameter *α* ∈ {0,0.4,1} and planning horizon *P* ∈ {0,2,7}.

### Level −1 and POMCP Rollout Mechanism

The level −1 models are obtained by having the level −1 agent always assume all partner types to be equally likely ($\mathbb{P}[\alpha^{\text{partner}}=\alpha_{i}]=\frac{1}{3},\forall i$), setting the planning horizon to 0, meaning the partner acts on immediate utilities only, and calculating the agent’s expected utilities after marginalizing over partner types and their respective response probabilities based on their immediate utilities.

In the POMCP treatment of the multi round trust game, if a simulated agent reaches a given history for the first time, a value estimate for the new node is derived by treating the agent as level −1 and using an *ε*-greedy decision making mechanism on the expected utilities to determine their actions until the present planning horizon.

### Behavioural Results

We adapted the POMCP algorithm [31] to solve IPOMDPs [23], and cast the multi-round trust task as an IPOMDP that could thus be solved. We made a number of approximations that were prefigured in past work in this domain [22, 24], and also made various observations that dramatically simplified the task of planning, without altering the formal solutions. This allowed us to look at longer planning horizons, which is important for the full power of the intentional modeling to become clear.

Here, we first seek to use this new and more powerful planning method to understand the classes of behaviour that arise from different settings of the parameters, as shown in the following section. From the study of human interactions [16], the importance of coaxing (returning more than the fair split) has been established. From our own study of the data collected so far, we define four coarse types of ‘pure’ interactions, which we call “Cooperation”, “Coaxing to Cooperation”, “Coaxing to Exploitation”, “Greedy”; we conceptualize how these might arise. We also delimit the potential consequences of having overly restricted the planning horizon in past work in this domain, and examine the qualitative interactive signatures (such as how quickly average investments and repayments rise or fall) that might best capture the characteristics of human subjects playing the game.

We then continue to discuss the quality of statistical inference, by carrying out model inversion for our new method and comparing to earlier work in this domain [24].

Finally, we treat real subject data collected for an earlier study ([22]) and show that our new approach recovers significant behavioural differences not obtained by earlier models and offers a significant improvement in the classification of subject behaviour through the inclusion of the planning parameter in the estimation and the quality of estimation on the trustee side.

### Modalities

All simulations were run on the local cluster at the Wellcome Trust Centre for Neuroimaging. For sample paths and posterior distributions, for each pairing of investor guilt, investor sophistication and trustee guilt and trustee sophistication, 60 full games of 10 exchanges each were simulated, totaling 8100 games. Additionally, in order to validate the estimation, a uniform mix of all parameters was used, implying a total of 2025 full games.

To reduce the variance of the estimation, we employed a pre-search method. Agents with ToM greater than 0 first explored the constant strategies (offering/returning a fixed fraction) to obtain a minimal set of $\tilde{Q}$ values from which to start searching for the optimal policy using SoftUCT. This ensures that inference will not “get stuck” in a close-to-optimal initial offer just because another initial offer was not adequately explored. This is more specific than just increasing the exploration bonus in the SoftUCT rule, which would diffuse the search during all stages, rather than helping search from a stable initial grid.

We set a number *n* of simulations for the initial step, where the beliefs about the partner are still uniform and the time horizon is still furthest away. We then reduce the number of simulations as the time horizon approaches $\left(n,n\frac{9}{10},n\frac{8}{10},\ldots ,n\frac{1}{10}\right)$.

### Simulation and Statistical Inference

Unless stated otherwise, we employ an inverse temperature in the softmax of $\beta =\frac{1}{3}$ (noting the substantial scale of the rewards). The exploration constant for POMCP was set to *c* = 25. The initial beliefs were uniform *a*_*i*_ = 1,∀*i*, for each subject. For the 3 possible guilt types we use the following expression while in text: *α* = *α*_1_ is “greedy”, *α* = *α*_2_ is “pragmatic” and *α* = *α*_3_ is “guilty”. However, on all the graphs, we give the exact model classification in the form *I*:(*k* ^*I*^, *α* ^*I*^, *P* ^*I*^) for the investor and *T*:(*k* ^*T*^, *α* ^*T*^, *P* ^*T*^) for the trustee.

We present average results over multiple runs generated stochastically from each setting of the parameter values. In the figures, we report the *actual* characteristics of investor and trustee; however, in keeping with the overall model, although each agent knows their own parameters, they are each inferring their opponents’ degree of guilt based on their initial priors.

As a consequence of our earlier observation in theorem 2, we only consider *k* ∈ {0,2} for the investor and *k* ∈ {0,1} for the trustee. Planning horizons are restricted to *P* ∈ {0,2,7}, as noted before, with the level 0 trustee always having a planning horizon of 0.

Actions for both agents are parametrized as in section “The Trust Task” and averaged across identical parameter pairings. In the graphs, we show actions in terms of the percentages of the available points that are offered or returned. For the investor, the numerical amounts can be read directly from the graphs; for the trustee, these amounts depend on the investor’s action. In the figures, we report the *actual* characteristics of investor and trustee; however, in keeping with the overall model, although each player knows their own parameters, they are each inferring their opponents’ degree of guilt based on their initial priors.

Dual to generating behaviour from the model is to invert it to find parameter settings that best explain observed interactions [22, 24]. Conceptually, this can be done by simulating exchanges between partners of given parameter settings (*k*, *α*, *P*), taking the observed history of investments and responses, and using a maximum likelihood estimation procedure which finds the settings for both agents that maximise the chance that simulated exchanges between agents possessing those values would match the actual, observed exchange. We calculate the action likelihoods through the POMCP method outlined earlier and accumulate the negative log likelihoods, looking for the combination that produces the smallest negative loglikelihood. This is carried out for each combination of guilt and sophistication for both investor and trustee.

### Paradigmatic Behaviours

The following figures show the three characteristic types of behaviour, in each case for two sets of parameters for investor and trustee. The upper graphs show the average histories of actions of the investor (blue) and trustee (red) across the 10 rounds; the middle graphs show the mean posterior distributions over the three guilt parameters (0,0.4,1) as estimated by the investor and the lower graphs show the mean posterior distribution by the trustee (right) at four stages in the game (rounds 0, 3, 6 and 9). These show how well the agents of each type are making inferences about their partners.

**Fig 11:**
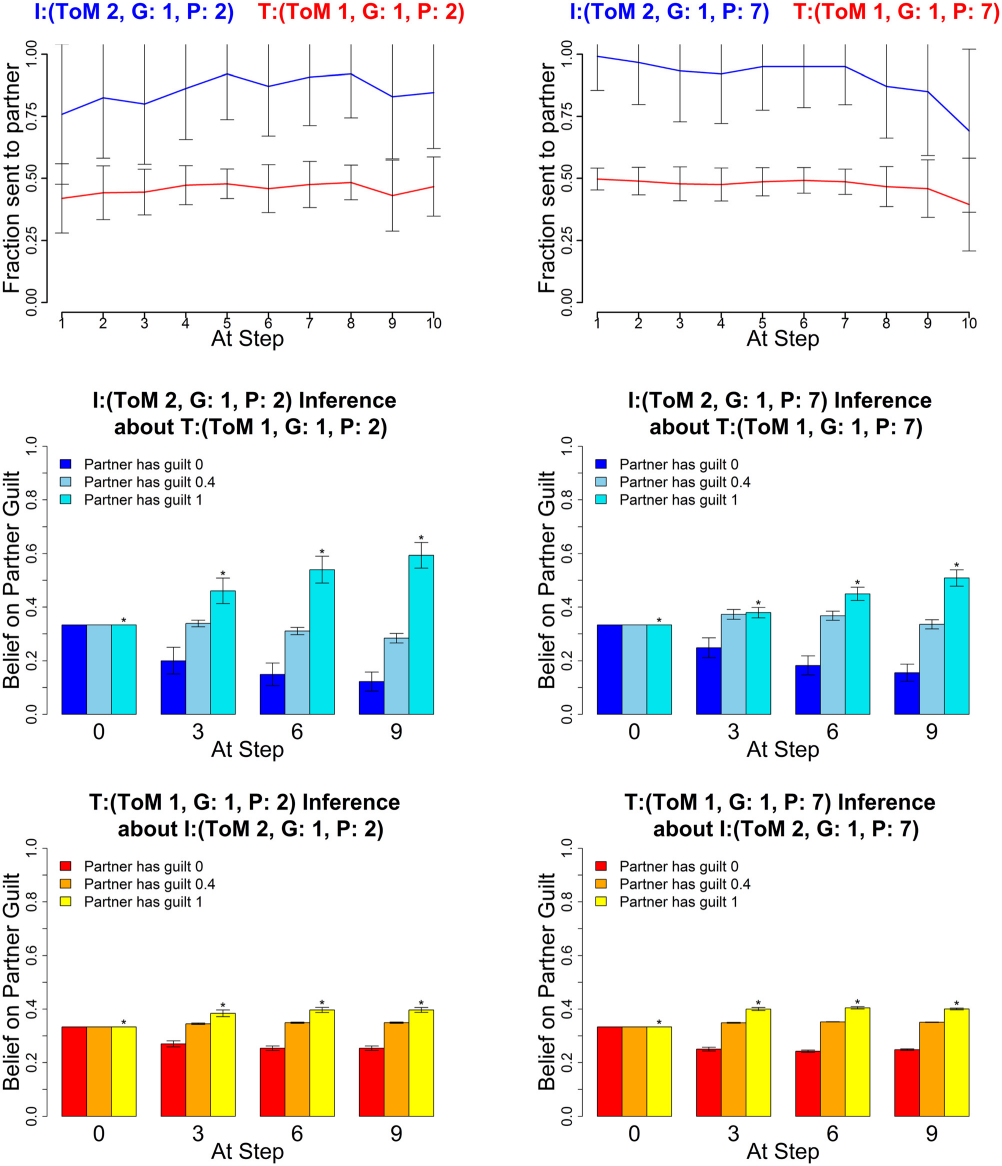
Guilty types. Averaged Exchanges (upper) and posteriors (mid and lower). Left plots: Investor (*k* ^*I*^, *α* ^*I*^, *P* ^*I*^) = (2,1,2); Trustee (1,1,2); right plots: Investor (2,1,7) and Trustee (1,1,7). The posterior distributions are shown for *α* = (0,0.4,1) at four stages in the game. Error bars are standard deviations. The asterisk denotes the true partner guilt value.

**Fig 12:**
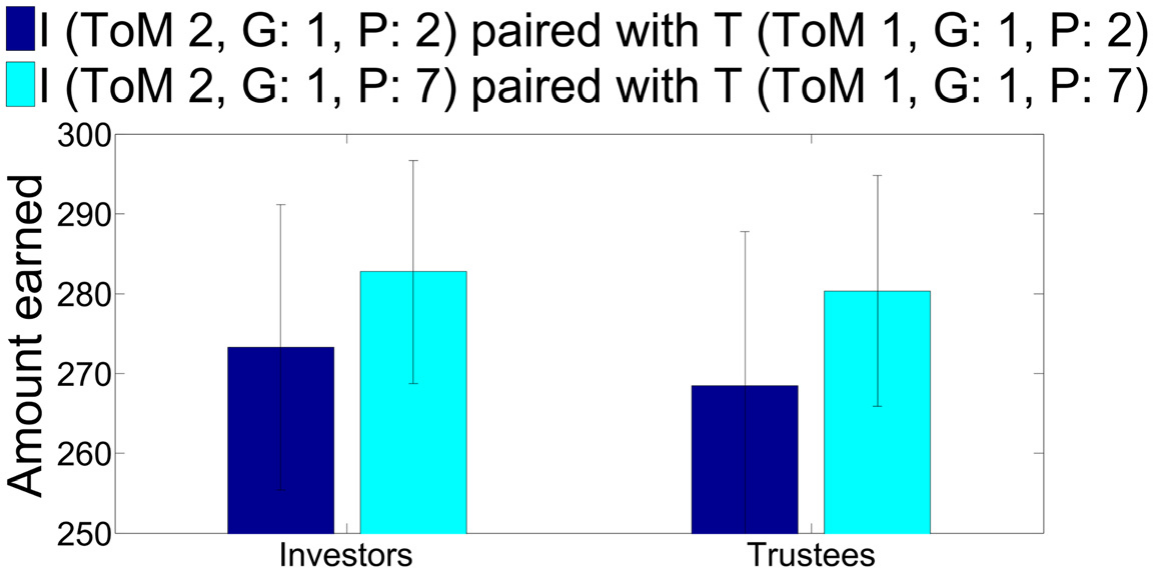
Average overall gains for the exchanges in Fig 11 with planning 2 (dark blue) and 7 (light blue). The difference is highly significant (*p* < 0.01) at a sample size of 60 for both parameter settings. Error bars are standard deviations.

Fig 11 shows evidence for strong cooperation between two agents who are characterized by high inequity aversion (i.e., guilty). Cooperation develops more slowly for agents with shorter (left) than longer (right) planning horizons, enabling a reliable distinction between different guilty pairs. This is shown more explicitly in Fig 12 in terms of the total amount of money made by both participants.

Both cases can be seen as cases of a tit for tat like approach by the players, although unlike a strict tit for tat mechanism the process leading to high level cooperation is generally robust against following below par actions by either player. Rather, high level players would employ coaxing to reinforce cooperation in this case. This is true even for lower level players, as after they have formed beliefs of the partner, they will not immediately reduce their offers upon a few low offers or returns, due to the Bayesian updating mechanism.

The posterior beliefs show both partners ultimately inferring the other’s guilt type correctly in both pairings, however the *P* ^*I*^ = 7 investors remain aware of the possibility that the partners may actually be pragmatic and therefore the high level long horizon investors are prone to reduce their offers preemptively towards the end of the game. This data feature was noted in particular in the study [22] and our generative model provides a generative explanation for it, based on the posterior beliefs of higher level agents explained above.

**Fig 13:**
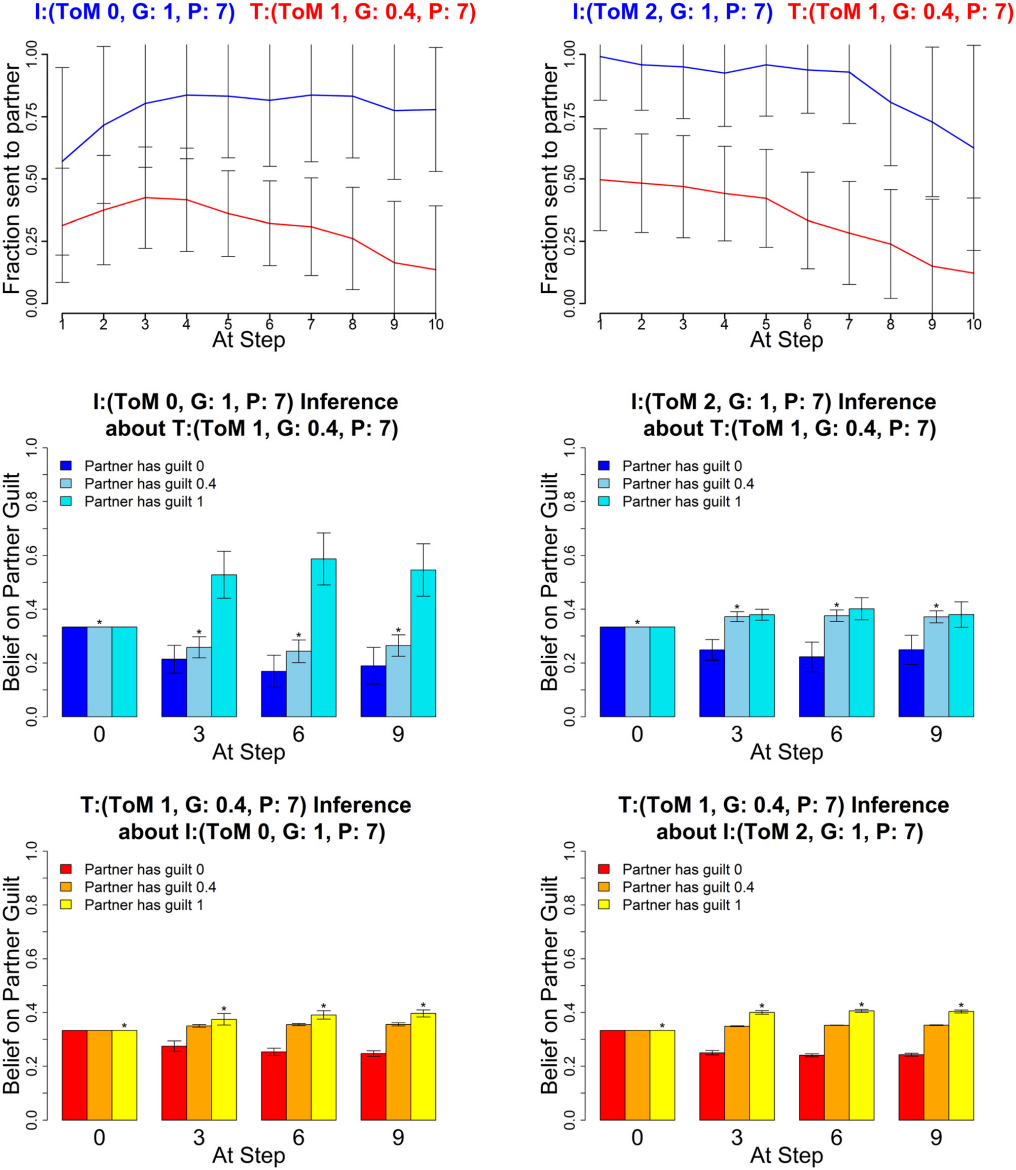
Deceptive trustees. Averaged Exchanges (upper) and posteriors (mid and lower). Left plots: Investor (*k* ^*I*^, *α* ^*I*^, *P* ^*I*^) = (0,1,7); Trustee (1,0.4,7); right plots: Investor (2,1,7) and Trustee (1,0.4,7). The posterior distributions are shown for *α* = (0,0.4,1) at four stages in the game. Error bars are standard deviations. The asterisk denotes the true partner guilt value.

Fig 13 shows that level 1 trustees employ coaxing (returning more than the fair split) to get the investor to give higher amounts over extended periods of time. In the example settings, the level 0 investor completely falls for the trustee’s initial coaxing (left), coming to believe that the trustee is guilty rather than pragmatic until towards the very end. However, the level 2 investor (right) remains cautious and starts reducing offers soon after the trustee gets greedy, decreasing their offers faster than if playing a truly guilty type. The level 2 investor on average remains ambiguous between the partner being guilty or pragmatic. Either inference prevents them from being as badly exploited as the level 0 investor.

In these plots, investor and trustee both have long planning horizons; we later show what happens when a trustee with a shorter horizon (*P* ^*T*^ = 2) attempts to deceive.

**Fig 14:**
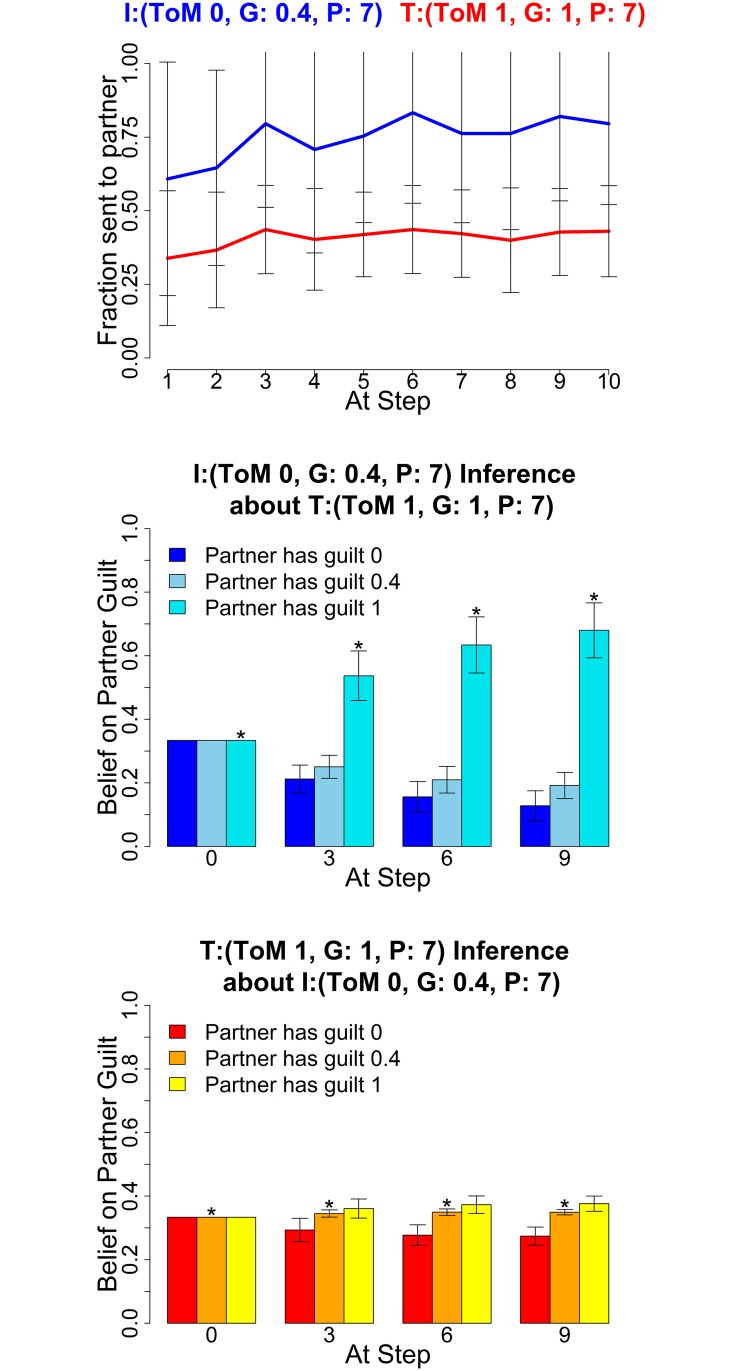
Driving up cooperation. Average Exchanges (upper) and posteriors (mid and lower), Investor (0,0.4,7) and Trustee (1,1,7). The posterior distributions are shown for *α* = (0,0.4,1) at four stages in the game. Error bars are standard deviations. The asterisk denotes the true partner guilt value.

A level 1 trustee can also get pragmatic investors to cooperate through coaxing, as demonstrated in Fig 14. The returns are a lot higher than for a level 0 guilty trustee, who lacks a model of their influence on the investor, and hence does not return enough to drive up cooperation. This initial coaxing is a very common behaviour of high level healthy trustees, trying to get the investor to cooperate more quickly, for both guilty and pragmatic high level trustees.

### Inconsistency or Impulsivity

**Fig 15:**
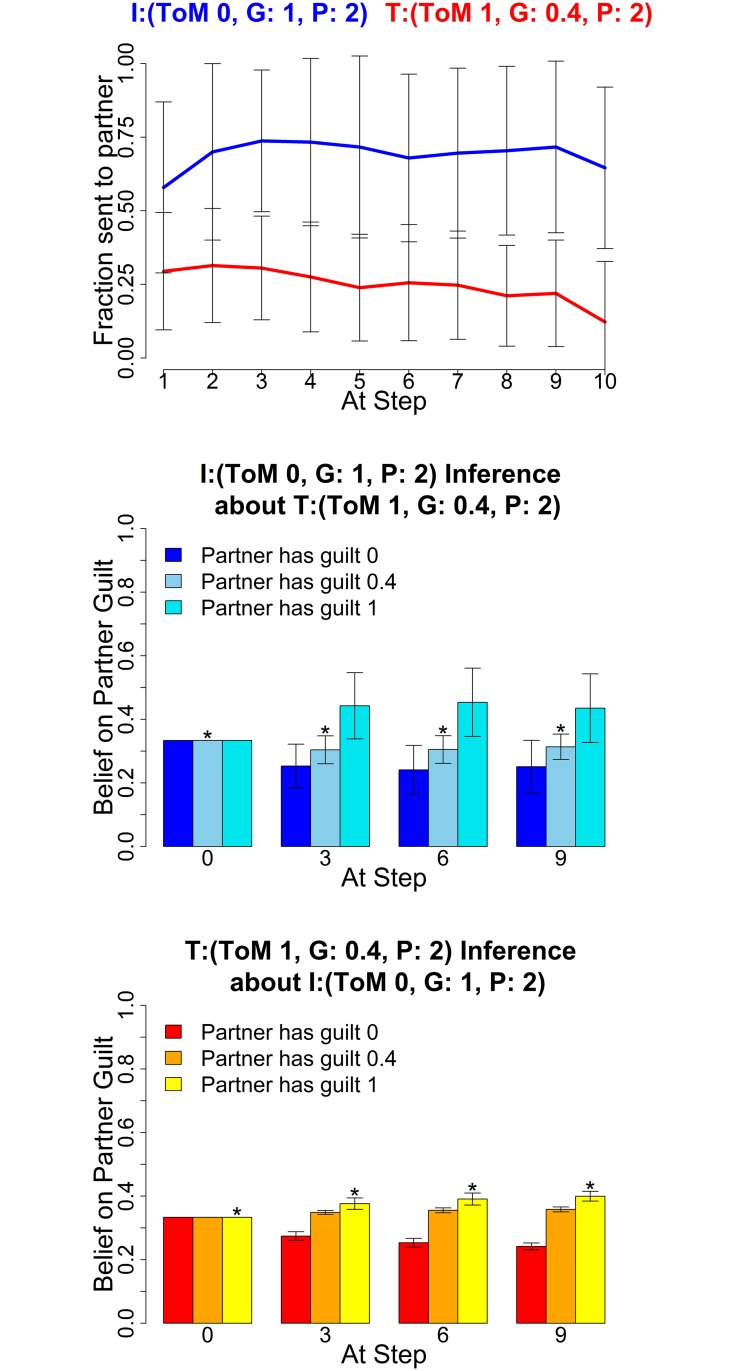
Impulsive trustee can not exploit consistently. Average Exchanges (upper) and posteriors (mid and lower), Investor (0,1,2) and Trustee (1,0.4,2). The posterior distributions are shown for *α* = (0,0.4,1) at four stages in the game. Error bars are standard deviations. The asterisk denotes the true partner guilt value.

Trustees with planning horizon 2 tend to find it difficult to maintain deceptive strategies. As can be seen in Fig 15, even when both agents have a planning horizon of 2, a short sighted trustee builds significantly less trust than a long sighted one. This is because it fails to see sufficiently far in the future, and exploits too early. This planning horizon thus captures cognitive limitations or impulsive behaviour, while the planning horizon of 7 generally describes the consistent execution of a strategy during play. Such a distinction may be very valuable for the study of clinical populations suffering from psychiatric disorders such as attention deficit hyperactivity disorder (ADHD) or borderline personality disorder (BPD), who might show high level behaviours, but then fail to maintain them over the course of the entire game. Inferring this requires the ability to capture long horizons, something that had eluded previous methods. This type of behaviour shows how important the availability of different planning horizons is for modeling, as earlier implementations such as [24] would treat this impulsive type as the default setting.

### Greedy Behaviour

**Fig 16:**
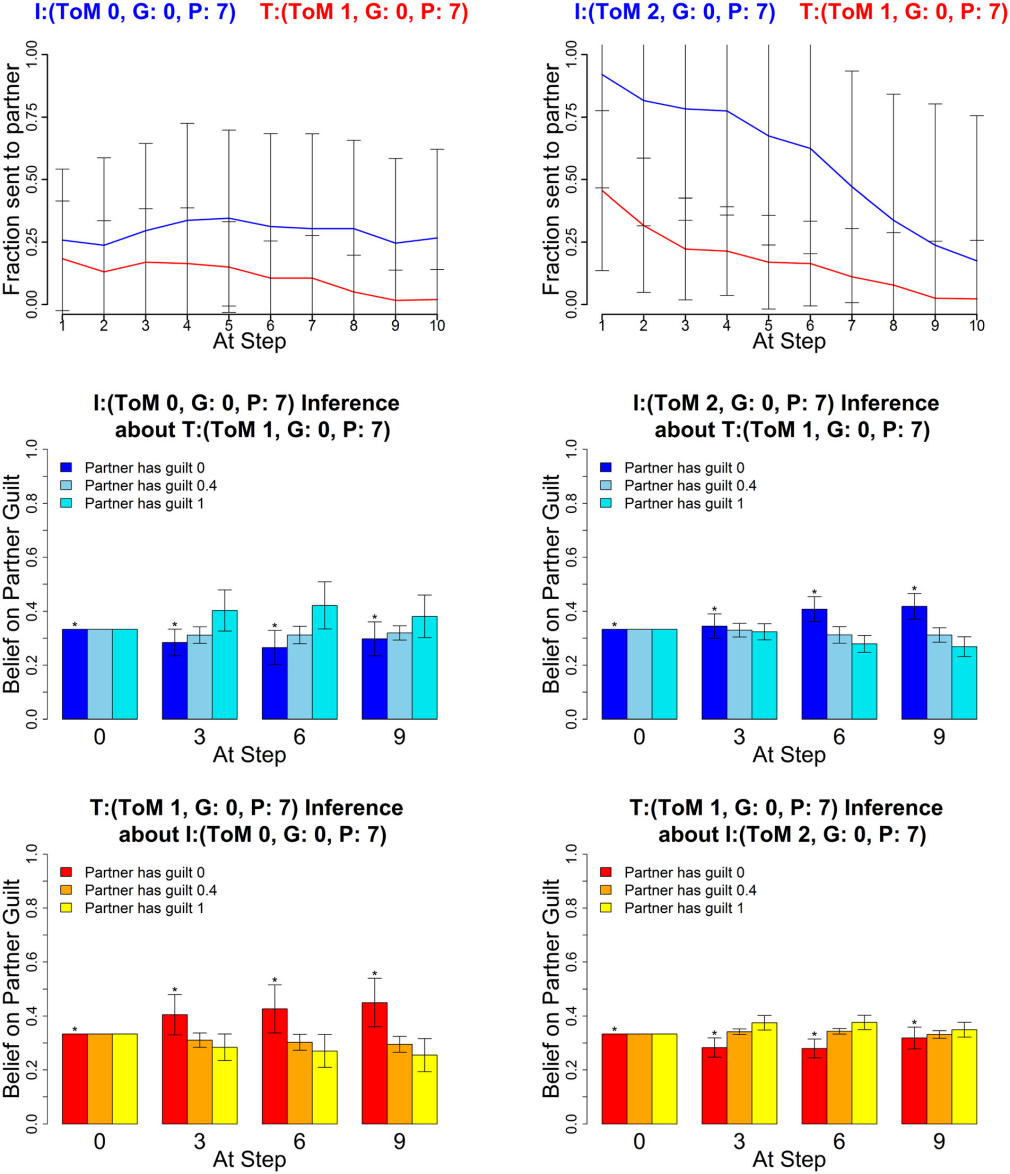
Greedy agents break cooperation. Averaged Exchanges (upper) and posteriors (mid and lower). Left plots: Investor (*k* ^*I*^, *α* ^*I*^, *P* ^*I*^) = (0,0,7); Trustee (1,0,7); right plots: Investor (2,0,7) and Trustee (1,0,7). The posterior distributions are shown for *α* = (0,0.4,1) at four stages in the game. Error bars are standard deviations. The asterisk denotes the true partner guilt value.

Another behavioural phenotype with potential clinical significance arises with fully greedy partners, see Fig 16. Greedy low level investors only invest very little, even if trustees try to convince them of a high guilt type on their part as described above (coaxing). Cooperation repeatedly breaks, which is reflected in the high variability of the investor trajectory. Two high level greedy types initially cooperate, but since the greedy trustee egregiously over-exploits, cooperation usually breaks down quickly over the course of the game, and is not repaired before the end. In the present context, the greedy type appears quite pathological in that they seem to hardly care at all about their partner’s type. The main exception to this is the level 2 greedy investor (an observation that underscores how theory of mind level and planning can change behaviour that would seem at first to be hard coded in the inequality aversion utility function). The level 0 greedy investor will cause cooperation to break down, regardless of their beliefs, as in Fig 16 the posterior beliefs of the level 0 show that they believe the trustee to be guilty, but do not alter their behaviour in the light of this inference.

### Planning Mismatch—High Level Deceived by Lower Level

**Fig 17:**
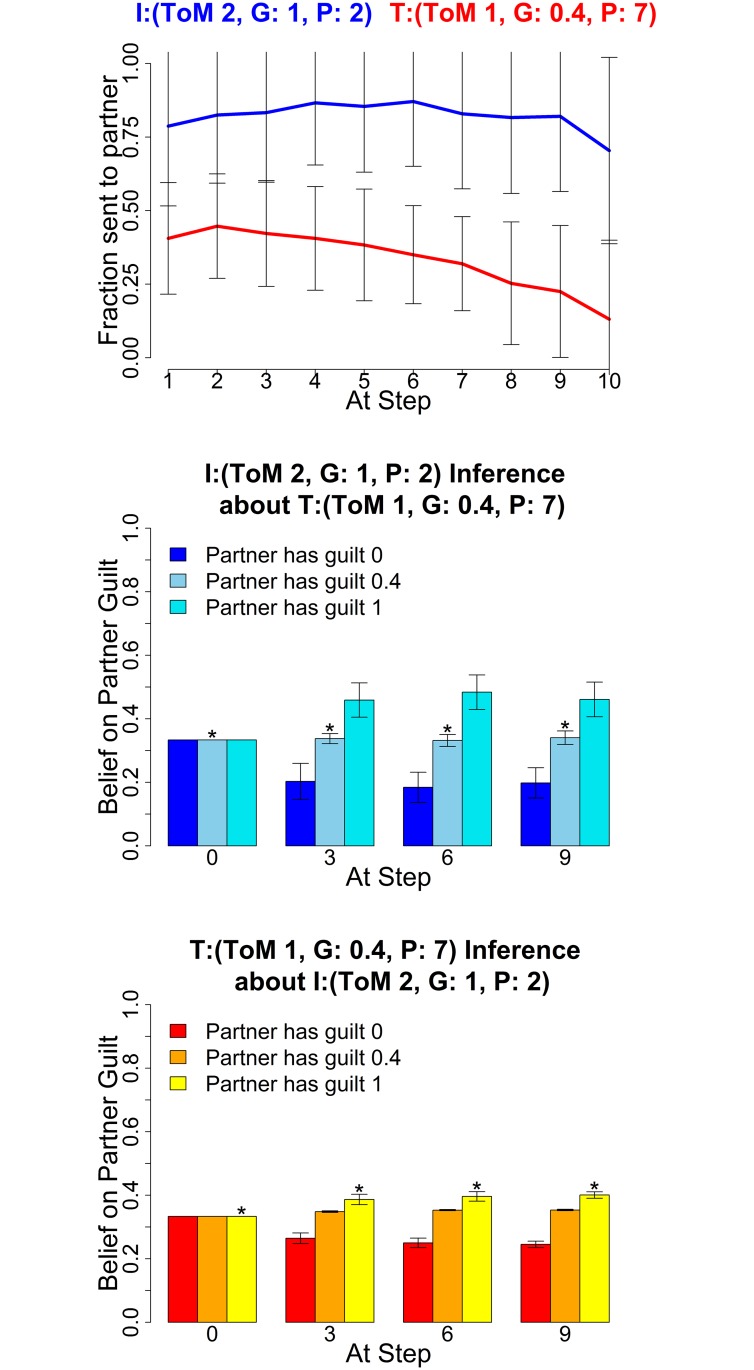
Higher level investor deceived by consistent trustee. Average Exchanges, Investor (2,1,2) and Trustee (1,0.4,7). Error bars are standard deviations. The asterisk denotes the true partner guilt value.

In Fig 17, the investor is level 2, and so should have the wherewithal to understand the level 1 trustee’s deception. However, the trustee’s longer planning horizon permits her to play more consistently, and thus exploit the investor for almost the entire game. This shows that the advantage of sophisticated thinking about other agents can be squandered given insufficient planning, and poses an important question about the efficient deployment of cognitive resources to the different demands of modeling and planning of social interactions.

### Confusion

#### Model inversion

A minimal requirement for using the proposed model to fit experimental data is self-consistency. That is, it should be possible to recover the parameters from behaviour that was actually generated from the model itself. This can alternatively be seen as a test of the statistical power of the experiment—i.e., whether 10 rounds suffice in order to infer subject parameters. We show the confusion matrix which indicates the probabilities of the inferred guilt (top), ToM (middle) and planning horizon (bottom) for investor (left) and trustee (right), in each case marginalizing over all the other factors. Afterwards, we discuss a particular special case of the obtained confusion. Said confusion relates to observations made in empirical studies (see [20, 22]) and suggests the notion of the planning parameter, as measure of consistency of play. Later, we show comparative data reported in the study [24], which only utilized a fixed planning horizon of 2 and 2 guilt states and did not exploit the other simplifications that we introduced above. These simplifications implied that the earlier study would find recovery of theory of mind in particular to be harder.

**Fig 18:**
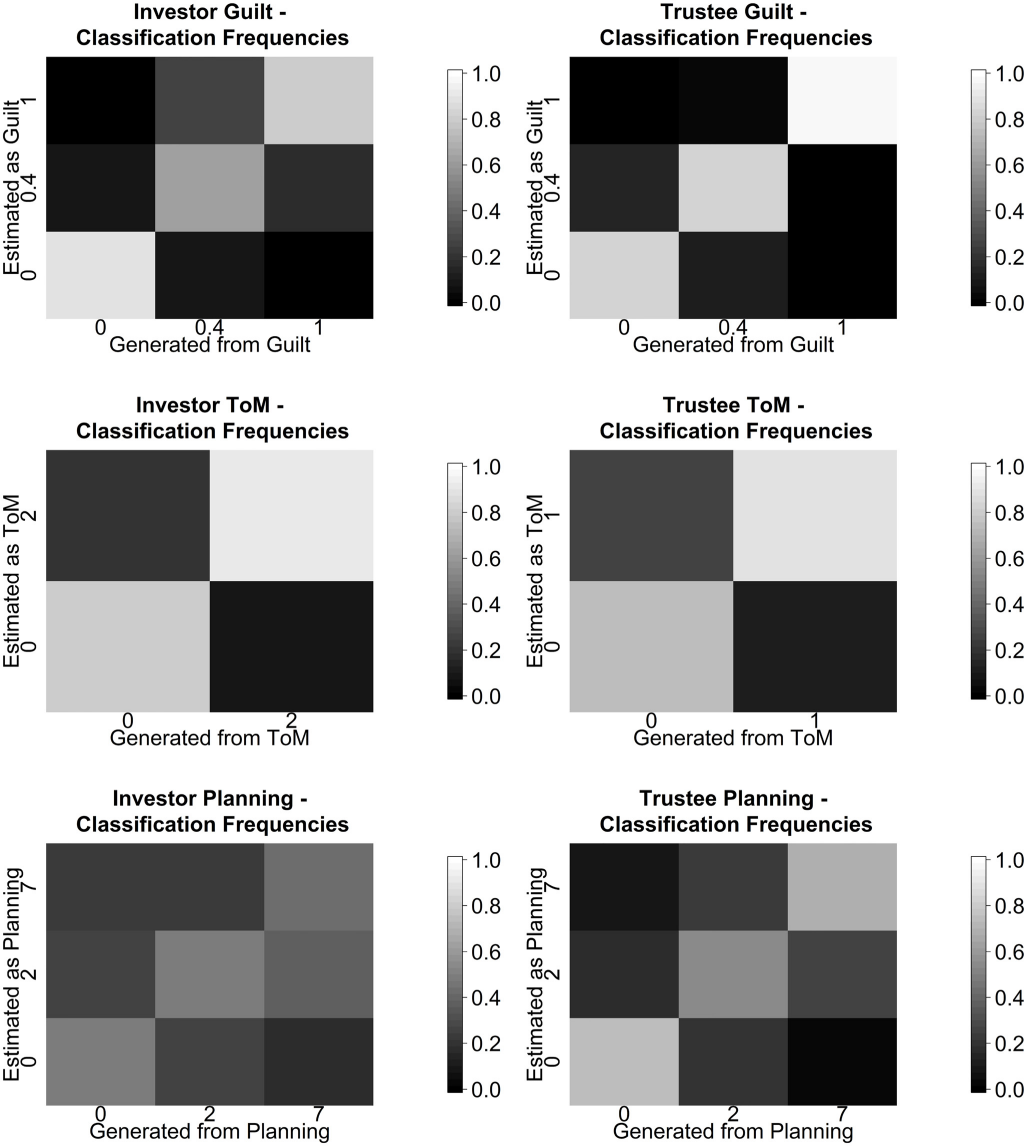
Percentage of inferred guilt, theory of mind and planning horizon for investor (left) and trustee (right) as a function of the true values, marginalizing out all the other parameters. Each plot corresponds to a uniform mix of 15 pairs per parameter combination and partner parameter combination.

As Fig 18 shows, Guilt is recovered in a highly reliable manner. By contrast, there is a slight tendency to overestimate ToM in the trustees.

**Fig 19:**
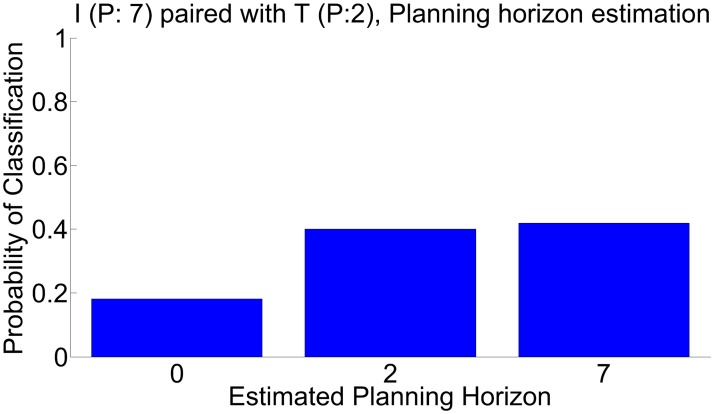
Planning misclassification. Maximum likelihood estimation result, *P* ^*I*^ = 7 and *P* ^*T*^ = 2 agent combinations, marginalized maximum likelihood estimation of investor planning horizon over all other parameters.

The greatest confusion turns out to be inferring a *P* ^*I*^ = 7 investor as having *P* ^*I*^ = 2 when playing an impulsive trustee (*P* ^*T*^ = 2), a problem shown more directly in Fig 19.

The issue is that when the trustee is impulsive, far-sighted investors (*P* ^*I*^ = 7) can gain no advantage over near-sighted ones (*P* ^*I*^ = 2), and so the choices of this dyad lead to mis-estimation. Alternatively put, an impulsive trustee brings the investor down to his or her level. This has been noted in previous empirical studies, notably [20, 22]’s observations of the effect on investors of playing erratic trustees. The same does not apply on the trustee side, since the reactive nature of the trustee’s tactics makes them far less sensitive to impulsive investor play.

Given the huge computational demands of planning, it seems likely that investors could react to observing a highly impulsive trustee by reducing their own actual planning horizons. Thus, the inferential conclusion shown in Fig 19 may in fact not be erroneous. However, this possibility reminds us of the necessity of being cautious in making such inferences in a two-player compared to a one-player setting.

#### Confusion comparison to earlier work

**Fig 20:**
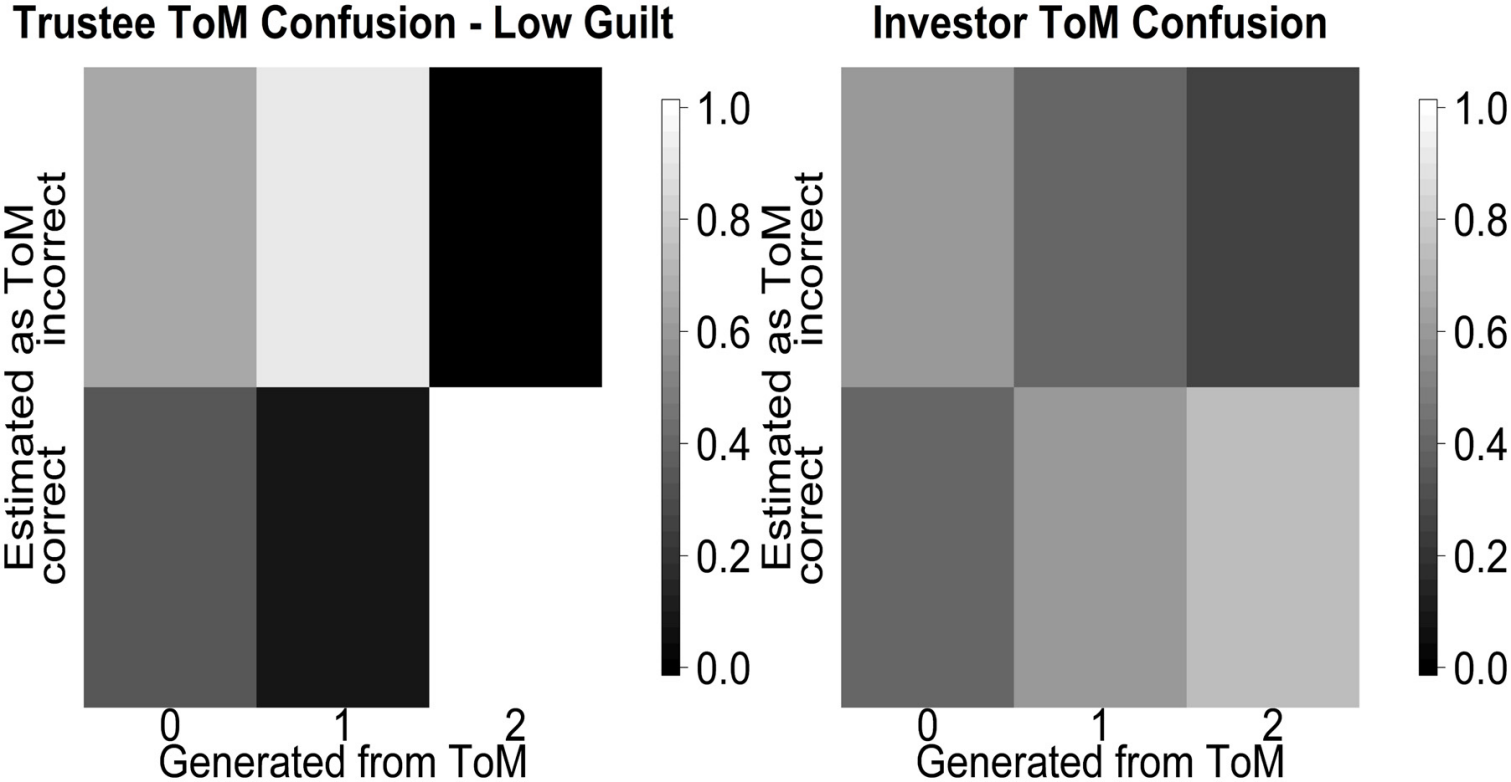
Classification probability reported in [24]. In analogy to Fig 18 we depict the generated vs estimated values in a matrix scheme.

We compare our confusion analysis to the one carried out in the grid based calculation in [24]. In [24] the authors do not report exact confusion metrics for the guilt state, only noting that it is possible to reliably recover whether a subject is characterized by high guilt (0.7) or low guilt (0.3). We can however compare to the reported ToM level recovery. The comparison with [24] faces an additional difficulty in that despite using the same formal framework as this present work, the indistinguishability of the level 1 and 2 trustees and the level 0 and 1 investors was not identified yet. This explains the somewhat higher amount of confusion when classifying ToM levels, reported in [24]. Also, since calculation of the Dirichlet-Multinomial probability was done numerically in this study, some between level differences will only derive from changes in quadrature points for higher levels. As can be seen in Fig 20 (left), almost all of the level 1 trustees at low guilt are misclassified. This is due to them being classified as level 2 instead, since both levels have the same behavioral features, but apparently the numerical calculation of the belief state favored the level 2 classification over the level 1 classification. The tendency to overestimation is true on the investor side as well, with there being a considerable confusion between level 0 and level 1 investors, who should behaviorally be equivalent. In sum, this leads to the reported overestimation of the theory of mind level. We have depicted the confusion levels reported in [24] in Fig 20.

### Computational Issues

**Fig 21:**
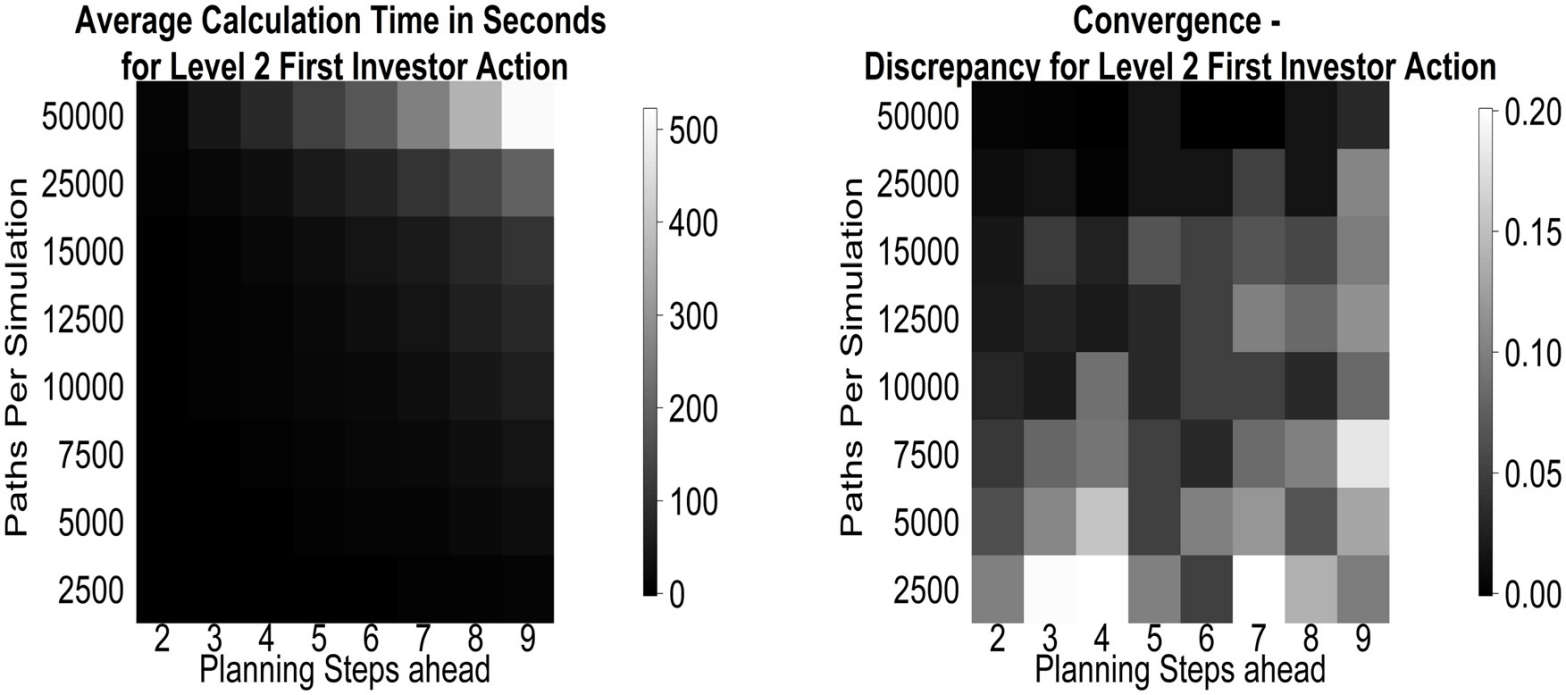
Numerical properties. (left) Average running times for calculating the first action value of a level 2, guilt 1 investor from a given number of simulations, as a function of planning horizon (complexity). (right) Discrepancy to the converged case of the action probabilities for the first action measured in squared discrepancies.

The viability of our method rests on the running time and stability of the obtained behaviours. In Fig 21, we show these for the case of the first action, as a function of the number of simulation paths used. All these calculations were run at the local Wellcome Trust Center for Neuroimaging (WTCN) cluster. Local processor cores where of Intel Xeon E312xx (Sandy Bridge) type clocked at 2.2 GHz and no process used more than 4 GB of RAM. Note that, unless more than 25*k* paths are used, calculations take less than 2 minutes.

We quantify simulation stability by comparing simulations for 120 level 2 investors (a reasonable upper bound, because the action value calculation for this incorporates the level 1 trustee responses) based on varying numbers of paths with a simulation involving 10^6^ paths that has converged. We calculate the between (simulated) subject discrepancies *C* of the probabilities for the first action for *P* ^*I*^ ∈ {2,3,4,5,6,7,8,9}:

$$\begin{array}{c} C_{ij}=\frac{1}{119}\sum_{k=1}^{120}\left(|{\mathbb{P}}^{k}\left[a_{0}^{I}=\frac{i}{4}\right]-\hat{\mathbb{P}}\left[a_{0}^{I}=\frac{i}{4}\right]|\right)\left(|{\mathbb{P}}^{k}\left[a_{0}^{I}=\frac{j}{4}\right]-\hat{\mathbb{P}}\left[a_{0}^{I}=\frac{j}{4}\right]|\right)\;i,j\in \left\{0,\ldots ,4\right\} \end{array}$$ (20)

where $\hat{\mathbb{P}}[a_{0}^{I}=\frac{i}{4}]$ are the converged probabilities, and ${\mathbb{P}}^{k}[a_{0}^{I}]$ is the action likelihood of simulated subject *k*. If the sum of squares of the entries in the discrepancy matrix is low, then the probabilities will be close to their converged values.

As can be seen from Fig 21 (right), for 25k paths even planning 9 steps ahead agents have converged in their initial action probabilities, such that their action probabilities vary from the converged value by no more than about 0.1. However, note that this convergence is not always monotonic in either the planning horizon or the number of sample paths. The former is influenced by the differing complexity of preferences for different horizons—sometimes, actions are harder to resolve for short than long horizons. The latter is influenced by the initial pre-search using constant strategies.

**Fig 22:**
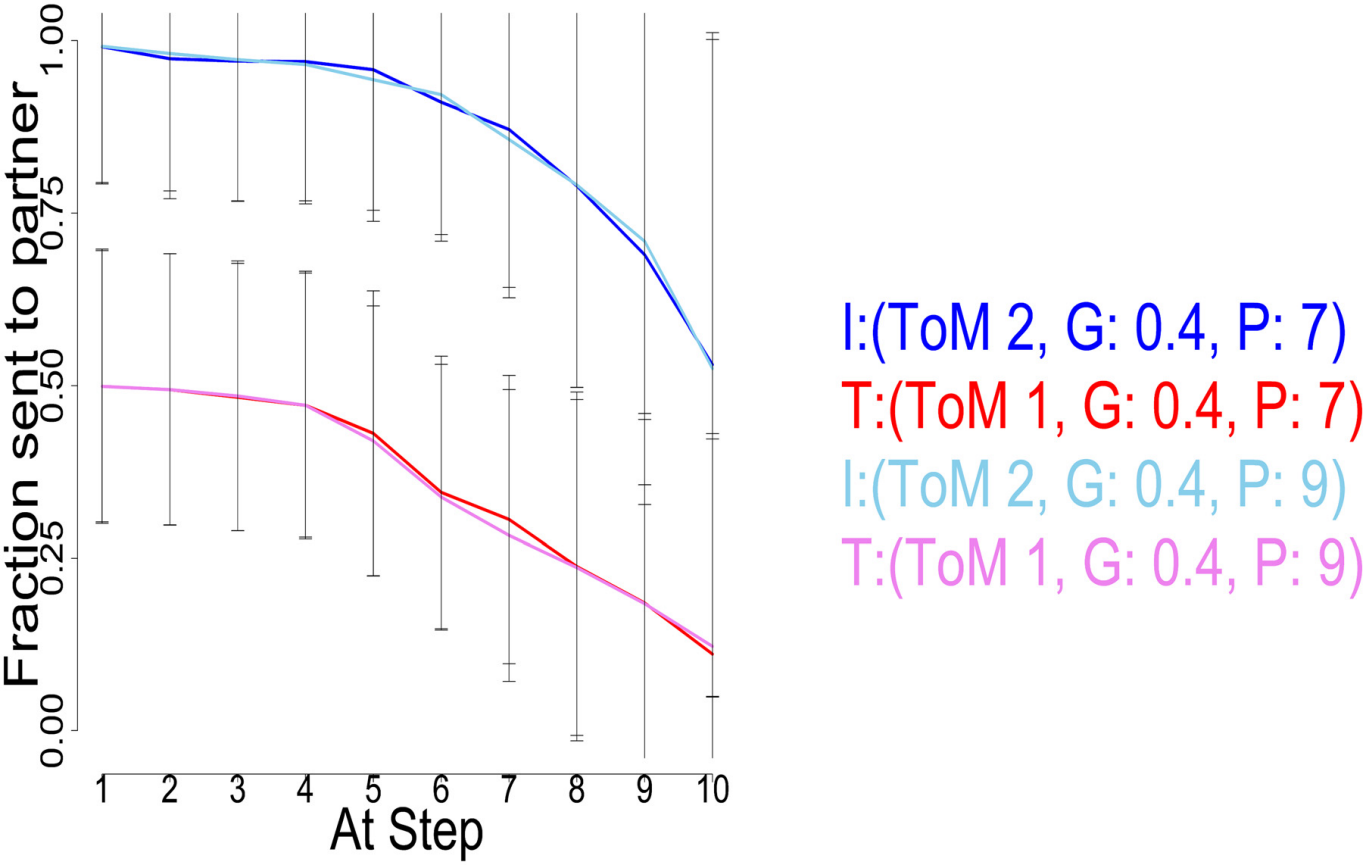
Planning horizon comparison. Average Exchanges, Investor (2,0.4,7) (dark blue) and Trustee (1,0.4,7) (red), as well as Investor (2,0.4,9) (light blue) and Trustee (1,0.4,9) (rose). The difference between the 2 planning horizons is not significant at any point. Error bars are standard deviations.

Although 25k steps suffice for convergence even when planning 9 steps ahead, this horizon remains computationally challenging. We thus considered whether it is possible to use a shorter horizon of 7 steps, without materially changing the preferred choices. Fig 22 illustrates that the difference is negligible compared with the fluctuations of the Monte Carlo approach, even for the worst case involving the pairing of 2 pragmatic types, with high ToM levels and long planning horizons. At the same time, the calculation for *P* = 7 is twice as fast as *P* = 9 for the level 2 investor, which even just for the first action is a difference of 100 seconds.

Finally we compare our algorithm at planning 2 steps ahead to the grid-based calculation used before [22, 24]. The speed advantage is a factor of 200 for 10^4^ paths in POMCP demonstrating the considerable improvement that enables us to consider longer planning horizons.

### Comparison To Earlier Subject Classifications

We will show below, using real subject data taken from [22], that our reduction to 3 guilt states does not render likelihoods worse and only serves to improve classification quality. We compared the results of our new method with the results obtained in earlier studes ([24], [22]).

#### Dataset

We performed inference on the same data sets as in Xiang et al, [22] (which were partially analysed in [24, 16] and [17]). This involved 195 dyads playing the trust game over 10 exchanges. The investor agent was always a healthy subject, the trustees comprised various clinical groups, including anonymous, healthy trustees (the “impersonal” group; 48 subjects), healthy trustees who were briefly encountered before the experiment (the “personal” group; 52 subjects), trustees diagnosed with Borderline Personality Disorder (BPD) (the “BPD” group; 55 subjects), and anonymous healthy trustees matched in socio-economic status (SES) to the (lower than healthy) SES distribution of BPD trustees, (the “low SES” group; 38 subjects).

#### Models used

We compared our models to the results of the model used in [22] on the same data set (which incorporates the data set used in [24]). The study [22] uses 5 guilt states {0,0.25,0.5,0.75,1} compared to our 3, a planning horizon of 2 and an inverse temperature of 1, otherwise the formal framework is exactly the same as in the section on the trust task. Action values in [22] were calculated by an exact grid search over all possible histories and a numerical integration for the calculation of the belief state. For comparison purposes we built a “clamped” model in which the planning horizon was fixed at the value 2, with 3 guilt states and a inverse temperature set to $\beta =\frac{1}{3}$. Additionally, we compared to the outcome for the full method in this work, including estimation of the planning horizon. We noted that in the analysis in [22], an additional approximation had been made at the level 0 investor level, which set those investors as non learning. This kept their beliefs uniform and yielded much better negative loglikelihoods within said model, than if they were learning.

#### Subject fit

A minimal requirement to accept subject results as significant is that the negative log likelihood is significantly better than random on average at *p* < 0.05, otherwise we would not trust a model based analysis over random chance and the estimated parameters would be unreliable. This criterion is numerically expressed as a negative loglikelihood of 16.1 for 10 exchanges, calculated from 5 possible actions at a probability of 0.2 each, with independent actions each round.

For the analysis in [22], we found that the level 0 approximation made in [22] allowed for significantly better negative investor log likelihoods (mean 11.98); if this approximation is removed, the investor data fit at an inverse temperature of 1 would be worse than random for this data set. Additionally, the model used in [22] did not fit the trustee data significantly better than random at *p* < 0.05 (mean negative loglikelihoods 15.6 and standard deviation of > 3).

Conversely, for both our clamped and full model analysis at $\beta =\frac{1}{3}$, the trustee likelihood is significantly better than random (11.7 at the full model) and the investor negative loglikelihood is slightly better on average (smaller) than found in [22] with 5 guilt states (11.7 for our method, vs 11.98). This confirms that reducing the number of guilt states to 3 only reduces confusion and does not worsen the fit of real subjects data. Additionally, it becomes newly possible to perform model-based analyses on the BPD trustee guilt state distribution, since the old model did not fit trustees significantly better than random at *p* < 0.05.

The seemingly low inverse temperature at $\beta =\frac{1}{3}$ is a consequence of the size of the rewards and the quick accumulation of higher expectation values with more planning steps, as the inverse temperature needs to counter balance the expectation size to keep choices from becoming nearly deterministic. Average investor outcome expectations (at the first exchange) for planning 0 steps stand at 18 with an average 18 being added at each planning step.

#### Marginal parameter distributions significant features

**Fig 23:**
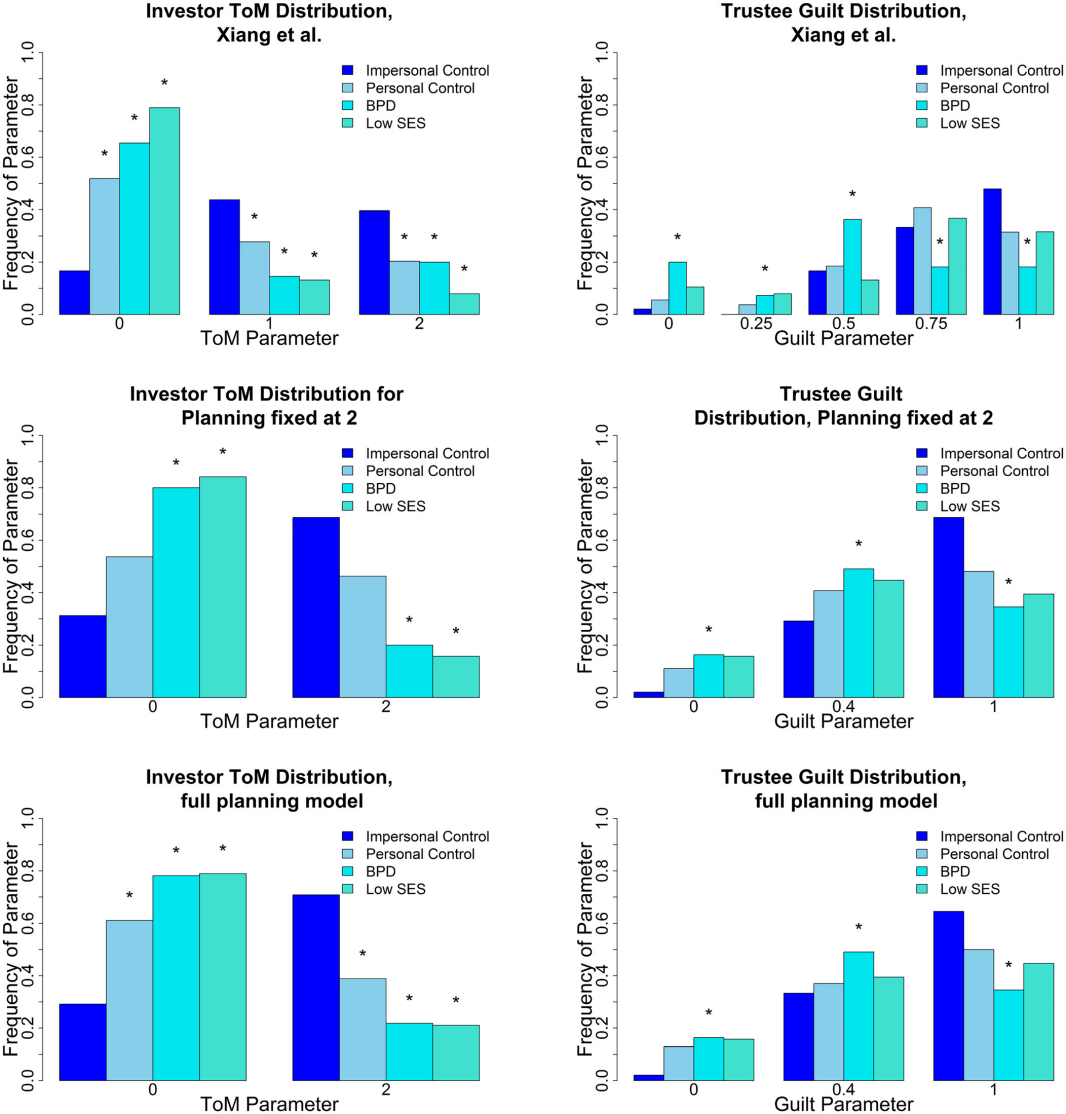
Parameter distributions for different models on the data set of [22]. (upper left) Investor ToM distribution is significant (*p* < 0.05) between the impersonal control condition and all other conditions. (upper right) Trustee Guilt distribution is significant between impersonal controls and the BPD trustees. (middle left) Planning 2 investor ToM distribution with 3 guilt states. BPD and low SES differences to impersonal are significant. (middle right) Planning 2 trustee guilt, the difference between BPD trustees and impersonal controls is significant. (bottom left) Full planning model investor ToM, all differences to impersonal are significant. (bottom right) Full planning model trustee guilt. BPD trustees are significantly different from controls. The asterisk denotes a significant (*p* < 0.05) difference in the Kolmogorov-Smirnov two sample test, to the impersonal control group.

Fig 23 shows the significant parameter distribution differences (Kolmogorov-Smirnov two sample test, *p* < 0.05).

**Fig 24:**
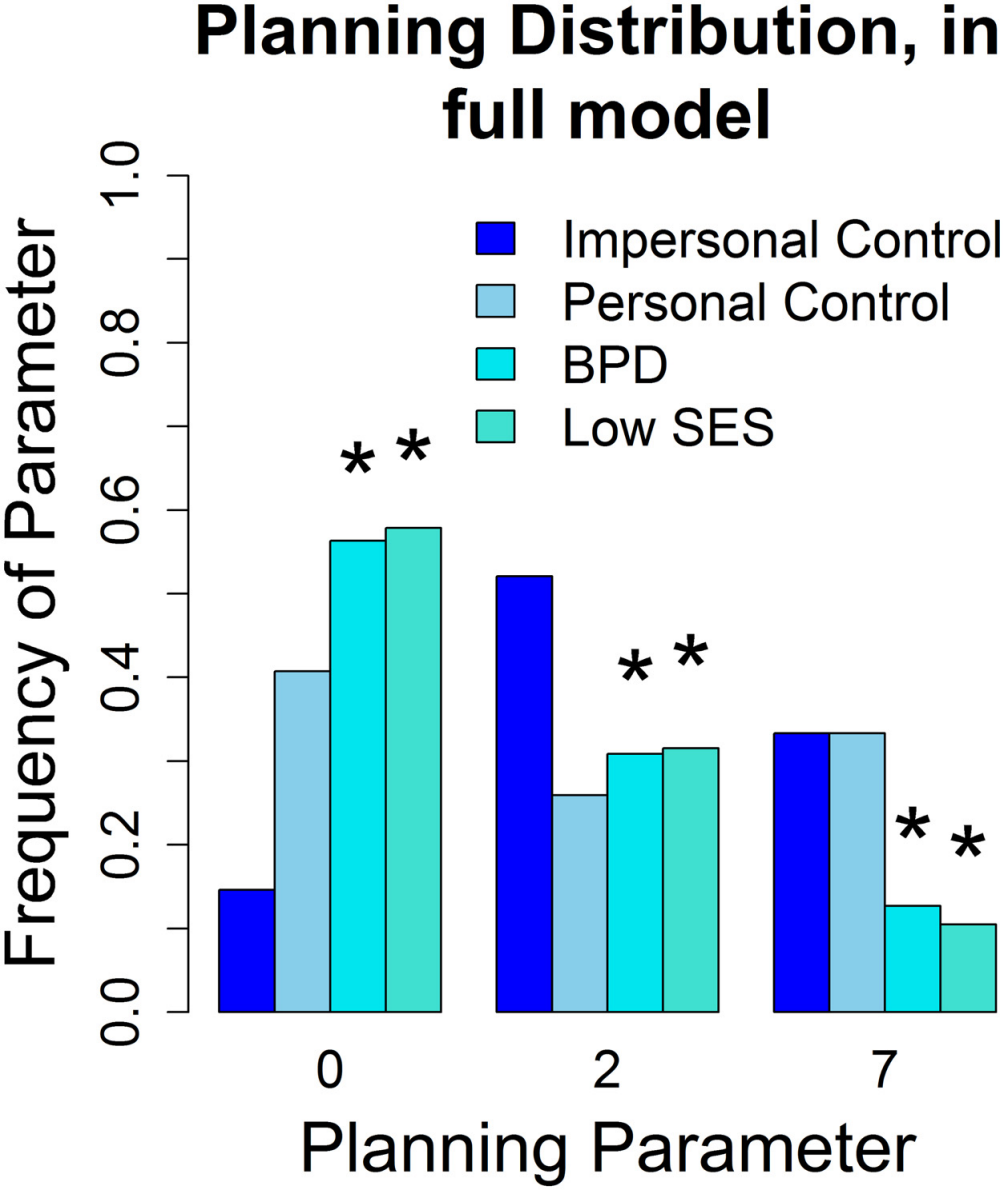
Planning horizon distribution on data set of [22]. Planning distribution for Investors, distinguished between personal condition controls (non significant) and BPD and low SES trustees (significantly lower than impersonal). The asterisk denotes a significant (*p* < 0.05) difference in the Kolmogorov-Smirnov two sample test, to the impersonal control group.

For investor theory of mind and trustee guilt distribution, many of the same differences are significant for the analysis reported in [22] (see Fig 23, upper panels), for an analysis using our model with a “clamped” planning horizon of 2 steps ahead (see Fig 23, middle panels, to match with the approach of [24]) and for our full model, using 3 guilt states, ToM level up to 2 and 3 planning horizons (see Fig 23, bottom panels and Fig 24). We find significantly lowered ToM in most other groups, compared to the impersonal control group. We find a significantly lowered guilt distribution in BPD trustees, however the guilt difference was not used for fMRI analysis in [22], because, as noted above, the trustee was not fit significantly better than random at *p* < 0.05 in the earlier model. For our full model with 3 planning values, we find additional significant differences on the investor side: While all ToM distributions are significantly different from the impersonal condition, the planning difference between the personal and impersonal conditions is not significant at *p* < 0.05, while it is significant for the other groups (see Fig 24). Thus, this is the only model keeping the parameter distribution of the personal group distinct from both the impersonal group (from which it is not significantly different in the clamped model) and the low SES playing controls and BPD playing controls (from which it is not significantly different based on the parameters in [22]) at the same time.

This supports the planning horizon as a “consistency of play” and additional rationality measure, as the subjects might not think about possible partner deceptions as much in the personal condition, having just met the person they will be playing (resulting in lowered ToM). However, their play is non disruptive, if lower level, and consistent exchanges result. BPD and low SES trustees however disrupt the partner’s play, lowering their planning horizon.

## Discussion

We adapted the Monte-Carlo tree search algorithm designed for partially observable Markov decision processes [31] to the interactive, game-theoretic, case [23]. We provide significant simplifications to the case of dyadic social exchange, which benefit any IPOMDP based method. We illustrated the power of this method by extending the computationally viable planning horizon in a complex, multi-round, social exchange game to be able to encompass characteristic behaviours that have been seen in human play [16].

We also showed that the 10 rounds that had been used empirically suffice to license high quality inference about parameter values, at least in the case that the behaviour was generated from the model itself. We exhibited three fundamental forms of dynamical behaviour in the task: cooperation, and two different varieties of coaxing. The algorithm generates values, state-action values and posterior beliefs, all of which can be used for such methods as model-based fMRI.

We find that the results in on impulsive behavior and planning mismatches, as well as Figs 19 and 24 confirm the planning horizon as a consistency of play parameter, that encodes the capability of a subject to execute a consistent strategy throughout play. As such it may be disrupted by the behavior of shorter planning partners, as can be seen in Fig 19 and Fig 24.

Furthermore, comparing to earlier data used in the work [22] we can confirm the relevance of the planning parameter in the treatment of real subject data, classifying subject groups along the new axis of consistency of play.

The newly finer classification of subjects along the three axes of theory of mind, planning horizon and guilt (*k*, *P*, *α*) should provide a rich framework to classify deficits in clinical populations such as an inability to model other people’s beliefs or intentions, ineffective model-based reasoning, and a lack of empathy. Such analyses can be done at speed, of the order of 10s of subjects per hour.

One might ask whether the behavioural patterns derived in this work might be obtained without invoking the cognitive hierarchy and instead using a large enough state space, which encodes the preferences and sophistication of the other agent as many separate states, rather than a few type parameters plus the cognitive hierarchy. This is in principle possible, however we prefer ToM for two reasons: Firstly, the previous study [22] and others have found neural support for the distinction between high ToM and low ToM subjects in real play, suggesting that this distinction is not but a mathematical convenience (cf. [22], p.4 and 5 for a neural representation of prediction errors associated to level 0 and level 2 thinking). Secondly, we can specify features of interest, such as inequality aversion and planning at the lowest level, then generate high level behaviours in a way that yields an immediate psychological interpretation in terms of the mentalization steps encoded in the ToM level.

The algorithm opens the door to finer analysis of complicated social exchanges, possibly allowing optimization over initial prior values in the estimation or the analysis of higher levels of theory of mind, at least on tasks with lower fan-out in the search tree. It would also be possible to search over the inverse temperature *β*.

One important lacuna is that although it is straightforward to use maximum likelihood to search over fixed parameters (such as ToM level, planning horizon or indeed temperature), it is radically harder to perform the computations that become necessary when these factors are incorporated into the structure of the intentional models. That is, our subjects were assumed to make inferences about their opponent’s guilt, but not about their theory of mind level or planning horizon.

It is possible that additional tricks would make this viable for the trust task, but it seems more promising to devise or exploit a simpler game in which this would be more straightforward.

## Materials and Methods

### Ethics Statement

Informed consent was obtained for all research involving human participants, and all clinical investigation was conducted according to the principles expressed in the Declaration of Helsinki. All procedures were approved by the Institutional Review Board of the Baylor College of Medicine.

### Code and Simulation Results

The materials used in this work, as well as the code used to generate them, can be found on Andreas Hula’s github repository https://github.com/AndreasHula/Trust. All material was generated on the local WTCN cluster. We used R [38] and Matlab [39] for data analysis and the boost C++ libraries [40] for code generation.
